# Integrated Untargeted and Targeted Metabolomics Reveals Distinct Bioactive Metabolite Profiles Between Probiotic Supplements and Yogurt

**DOI:** 10.3390/ijms27052180

**Published:** 2026-02-26

**Authors:** Sang Hyeon Noh, Su-Hyun Kim, Do Hoon Kwon, Choong Hwan Lee

**Affiliations:** Department of Bioscience and Biotechnology, Konkuk University, Seoul 05029, Republic of Korea; tkdgus131366@nate.com (S.H.N.); kimsuhyun2019@naver.com (S.-H.K.); cerisier123@naver.com (D.H.K.)

**Keywords:** probiotic supplements, yogurt, metabolomics, bioactive metabolites, antioxidant activity, anti-glycation activity, indole derivatives

## Abstract

Probiotics are widely consumed as health-promoting agents, with probiotic supplements (PS) and yogurt (YG) representing formulated products and fermented foods, respectively. Despite their broad consumption, systematic comparisons of their biochemical characteristics remain limited. In this study, integrated untargeted and targeted metabolomics approaches were applied to compare the comprehensive metabolite profiles of PS and YG. PS exhibited relatively higher levels of amino acids, dicarboxylic acids, and lysophospholipids, along with short-chain fatty acids such as acetate and propionate, and amino acid-derived bioactive metabolites, including *γ*-aminobutyric acid, branched-chain hydroxy acids, indole derivatives, and *γ*-glutamylpeptides. In contrast, YG showed higher relative abundances of carbohydrates, acylcarnitines, sphingolipids, and bioactive metabolites such as butyrate, creatine, carnitine, and orotic acid. Based on these metabolomic differences, 27 PS-specific and 17 YG-specific marker metabolites were identified. To explore their functional relevance, in vitro antioxidant and antiglycation activities were evaluated. PS exhibited significantly higher antioxidant and antiglycation activities than YG, which were positively correlated with amino acids and indole derivatives. Indole-3-acrylic acid, indole-3-acetic acid, and indole-3-propionic acid showed antiglycation activity and were identified as PS-specific bioactive marker metabolites. These findings reveal the distinct biochemical characteristics of PS and YG and highlight potential bioactive candidate metabolites that may contribute to their functional differences.

## 1. Introduction

Probiotics have gained increasing attention owing to their potential health benefits, and their global market has continued to expand. They are consumed in various forms, including fermented foods such as yogurt (YG), kefir, sauerkraut, and kimchi, which contain microbial cells embedded within a fermented food matrix, and formulated products such as probiotic supplements (PS) containing concentrated microbial cells [1]. Among them, YG and PS are the most widely consumed. These two products differ markedly in terms of their manufacturing and processing procedures. YG is typically produced by milk fermentation using lactic acid bacteria [2], whereas PS are manufactured by cultivating, isolating, and drying probiotic strains [3,4]. These differences in the manufacturing processes and substrate composition can influence the physiological and biochemical characteristics of the final products.

Several studies have reported distinct health outcomes associated with PS and YG. According to Yan et al. [5], PS might be more effective than fermented milk in relieving constipation. Conversely, YG consumption has been associated with a reduced risk of all-cause mortality and colorectal cancer compared with PS intake [6,7]. Emerging evidence indicates that microbial metabolites play a central role in mediating these health benefits [8]. For example, microbial-derived branched-chain hydroxy acids (BCHAs) have been proposed as potential contributors to the antidiabetic effects of YG [9]. Additionally, *γ*-aminobutyric acid (GABA), short-chain fatty acids (SCFAs), bile acids, indole derivatives, and vitamins are widely acknowledged as important microbial-derived metabolites with potential functional relevance [10,11]. Despite these insights, systematic metabolomic comparisons of PS and YG remain limited, leaving the metabolomic basis for their functional differences poorly understood.

Metabolomics is a comprehensive analytical field that characterizes low-molecular-weight compounds (<1500 Da) present in diverse biological samples and is broadly classified into untargeted and targeted approaches depending on the analytical purpose and scope. Untargeted metabolomics provides a wide and unbiased overview of metabolic profiles, making it particularly useful for identifying potential biomarkers associated with functional differences [12]. However, untargeted approaches may have limitations in the sensitive or accurate detection of the metabolites of interest. To overcome these limitations, targeted metabolomics has been employed to precisely monitor predefined metabolites with high analytical confidence [13]. In addition, owing to its high mass accuracy and resolving power, high-resolution mass spectrometry (HRMS) substantially improves the reliability of metabolite detection and identification in complex matrices.

Reactive oxygen species (ROS) are generated under oxidative stress, and their excessive accumulation has been linked to intestinal inflammation and the development of metabolic disorders [14]. Similarly, reactive dicarbonyl species such as methylglyoxal (MGO), generated during metabolic processes, react with amino groups of proteins to form advanced glycation end products (AGEs). These AGEs, formed through glycation reactions, have been reported to induce oxidative stress and inflammation and are associated with dysbiosis and metabolic disorders [15]. Therefore, ROS and AGEs are important indicators of pathological changes related to gut health. Therefore, identifying the metabolites associated with antioxidant and anti-glycation activities of probiotic products will provide important insights into the functional relevance of PS and YG.

Accordingly, this study aimed to compare the comprehensive metabolite profiles of PS and YG, incorporating targeted metabolomics for known bioactive metabolites. Antioxidant and anti-glycation activities were evaluated to investigate the potential functional associations of the observed metabolite differences. Our findings provide molecular insights into the functional properties of PS and YG.

## 2. Results and Discussion

### 2.1. Untargeted Metabolite Profiling of Probiotic Supplements and Yogurt

**Figure 1:**
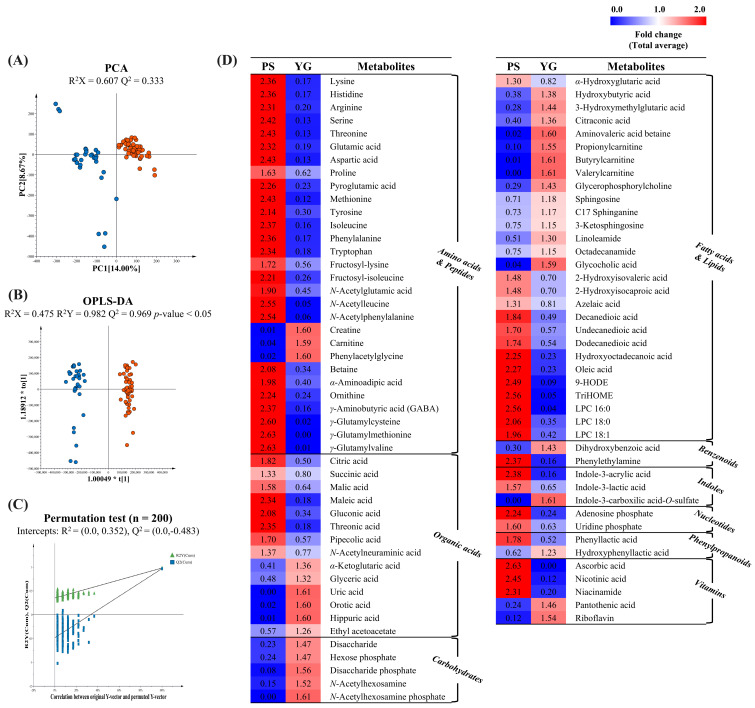
Untargeted metabolomic differences between probiotic supplements and yogurt. Score plots of (**A**) principal component analysis (PCA) and (**B**) orthogonal partial least squares discriminant analysis (OPLS-DA) comparing probiotic supplements (blue circles) and yogurt (orange circles). (**C**) 200 permutation test validating the OPLS-DA models. (**D**) Heat map of the relative abundance of discriminant metabolites based on the OPLS-DA model (VIP > 1.0; *p* < 0.05).

Multivariate statistical analysis was performed by integrating positive and negative data obtained from UHPLC–Orbitrap-MS/MS analysis of PS and YG. The QC samples clustered well in the PCA score plot, indicating that the data were reliable (Figure S1). The unsupervised PCA score plot revealed a clear separation between PS and YG (Figure 1A), with PC1 explaining 14% of the total variance, indicating distinct metabolomic differences. The relatively low cumulative variance likely reflects both the complexity of the samples and the compositional heterogeneity between PS and YG. To further investigate the contributions of different variables to this separation, supervised OPLS-DA was performed. The OPLS-DA score plot exhibited a similar separation pattern with statistical significance (*p* < 0.05) (Figure 1B). The OPLS-DA model was validated using a permutation test with 200 iterations, and the intercepts of R^2^ and Q^2^ were 0.352 and −0.483, respectively, indicating that a good predictive ability of the model without overfitting (Figure 1C). To identify metabolites that contributed to the differences between the PS and YG groups, discriminant metabolites were selected based on VIP > 1.0 and *p* < 0.05, derived from the respective OPLS-DA score plot and independent samples t-test. A total of 90 discriminant metabolites were identified, including 28 amino acids and peptides, 5 carbohydrates, 29 fatty acids and lipids, 14 organic acids, 2 benzenoids, 3 indoles, 2 nucleotides, 2 phenylpropanoids, and 5 vitamins (Table S2). The relative abundances of the discriminant metabolites between PS and YG are shown in Figure 1D.

PS exhibited a relatively higher abundance of several metabolite classes than YG, including amino acids, peptides, specific fatty acids, lipids, and nucleotides. Free amino acids and peptides have been reported to be produced and released during probiotic cultivation processes [16,17]. However, according to the product information (Table S1), PS ingredients, such as isolated soy proteins, L-arginine, and fermented grain powders, may also contribute to the observed metabolite profiles. Therefore, the metabolites enriched in PS may reflect not only microbial-derived components but also formulation-related constituents. Among the fatty acids and lipids, medium- and long-chain dicarboxylic acids (azelaic acid, decanedioic acid, undecanedioic acid, and dodecanedioic acid), oxylipins (9-HODE and TriHOME), and lysophospholipids (LPC 16:0, LPC 18:0, LPC 18:1, and LPG 14:0) were most abundant in PS.

By contrast, YG showed higher relative levels of carbohydrates, fatty acid–related metabolites including short-chain acylcarnitines (propionylcarnitine, butyrylcarnitine, and valerylcarnitine), and lipids such as sphingolipids (sphingosine, C17-sphinganine, and 3-ketosphingosine). The high abundance of carbohydrates in YG is likely attributable to milk-derived sugars, including lactose and its fermentation-related derivatives. Carnitine plays a central role in fatty acid transport into the mitochondria, whereas acylcarnitines buffer the intracellular free CoA pool and are essential for energy metabolism. Dairy products have been reported to naturally contain carnitine and acylcarnitines, which are compounds involved in lipid and energy metabolism, particularly in the context of early life, when the reliance on fat metabolism is high [18,19,20]. The higher abundance of sphingolipids observed in YG may be associated with milk fat globules, which are a major source of sphingolipids in dairy products [21,22]. In addition, microbial lysates have been reported to exhibit sphingomyelinase activity, which may contribute to the generation of sphingolipid-related metabolites from milk phospholipids during fermentation [10]. Furthermore, several organic acids, including orotic acid, hippuric acid, and uric acid, were relatively more abundant in YG. These organic acids are known constituents of milk [23,24,25], supporting their higher levels in YG. Overall, these results indicate that the biochemical characteristics of PS and YG may reflect differences in their composition.

### 2.2. Targeted Metabolomic Comparison of Bioactive Metabolites Between Probiotic Supplements and Yogurt

**Figure 2:**
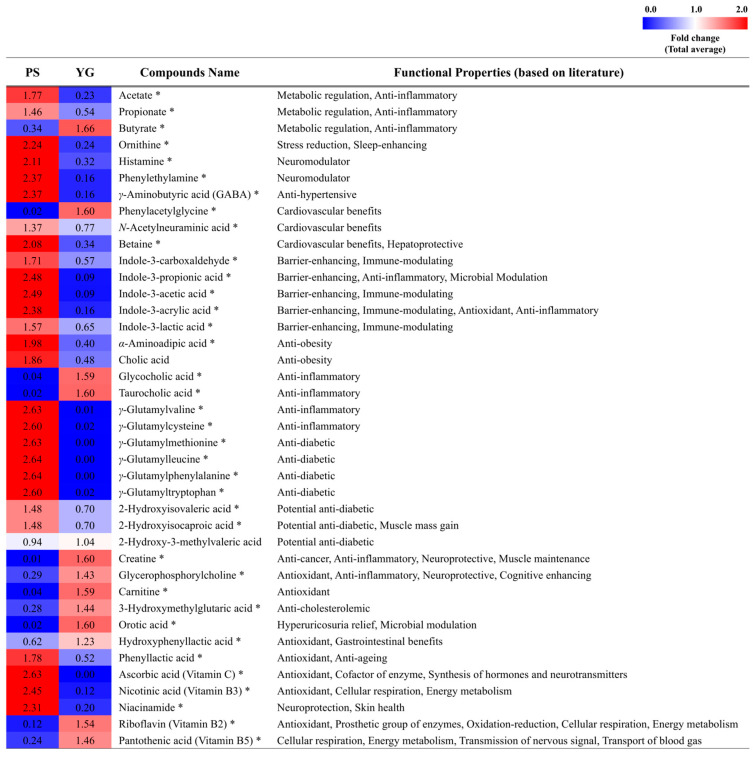
Comparison of the relative abundance of bioactive metabolites between probiotic supplements (PS) and yogurt (YG). Bioactive metabolites were identified using data based on UHPLC–Orbitrap-MS/MS and UHPLC–Triple Q-MS/MS. Detailed information on the functional properties of the literature-based bioactive metabolites is provided in Table S4. Compounds that show statistical significance (*p* < 0.05) between PS and YG are marked with an asterisk (*).

Untargeted metabolomics may overlook specific metabolites of interest, particularly those that are present at low concentrations or outside the optimal detection range. Therefore, to complement the untargeted approach, a targeted metabolomic comparison of microbially derived bioactive metabolites was performed. Based on literature evidence regarding their physiological relevance, 65 bioactive metabolites, including SCFAs, bile acids, indole derivatives, GABA, and vitamins, were selected as the target compounds (Table S3). Among these, 37 bioactive metabolites were successfully identified in the samples (Table S2), and their relative abundances were compared between PS and YG (Figure 2).

Compared with YG, PS showed relatively higher levels of amino acid–derived metabolites, including histamine, phenylethylamine, GABA, *γ*-glutamylpeptides, indole derivatives, and branched-chain hydroxy acids (BCHAs). Histamine, phenylethylamine, and GABA are produced during fermentation through the activity of microbial amino acid decarboxylases [26] and are involved in neuromodulation [27,28]. In addition, indole compounds derived from tryptophan metabolism have been reported to be associated with physiological functions such as intestinal barrier maintenance, modulation of inflammatory responses, and immune regulation [29,30,31]. Among the BCHAs, 2-hydroxyisovaleric acid and 2-hydroxyisocaproic acid were more abundant in PS, whereas 2-hydroxy-3-methylvaleric acid was slightly more abundant in YG (~1.11-fold). The relatively higher abundance of these amino acid-derived bioactive metabolites in PS may be associated with the higher amino acid content observed in PS (Figure 1D). By contrast, creatine was more abundant in YG than in PS. Creatine is a naturally occurring nitrogen-containing compound found in animal tissues [32] and has been widely studied for its potential health-related properties [33].

Growing interest in gut health has led to increased attention toward bile acids as important metabolites involved in host–microbiota interactions. In the present study, primary bile acids exhibited differences in conjugation patterns between PS and YG. The unconjugated bile acid, cholic acid, was more abundant in PS, whereas the conjugated bile acids, glycocholic acid and taurocholic acid, were more abundant in YG. Primary bile acids can be further transformed by gut microbial metabolism into various secondary bile acids, which have been reported to be associated with gut microbial composition and metabolic regulation [34].

SCFAs are primarily produced through the microbial fermentation of carbohydrates and play a central role in gut metabolic homeostasis. Among these SCFAs, acetate, propionate, and butyrate account for more than 95% of total SCFAs generated in the human gut [35]. However, reliable detection of SCFAs remains analytically challenging owing to their high polarity, volatility, and low molecular weight. Because the objective of this analysis was to compare the relative abundances of major SCFAs at the group level between PS and YG, targeted analysis was performed using pooled raw product samples. This approach enabled representative profiling of group-level SCFA composition; however, it limits the assessment of biological variability across individual products. Acetate and propionate were more abundant in PS than in YG, whereas butyrate was more abundant in YG. Previous reports indicating that a higher proportion of butyrate relative to acetate and propionate in milk provides contextual support for the relatively higher abundance of butyrate observed in YG [36]. Butyrate has received considerable attention owing to its association with immune regulation and colorectal cancer [37,38]. Collectively, the differences in potential bioactive metabolite composition may be related to variations in functional characteristics between PS and YG.

### 2.3. Determination of Specific Marker Metabolites of Probiotic Supplements and Yogurt

**Figure 3:**
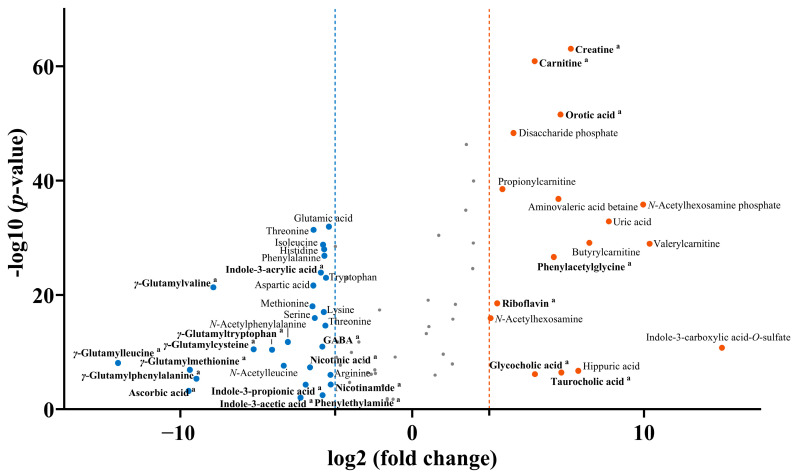
Volcano plot of marker metabolites differentiating probiotic supplements (PS) and yogurt (YG). Marker metabolites (blue: PS; orange: YG) were determined through integration of discriminant metabolites from untargeted analysis (VIP > 1.0, *p* < 0.05, and |p(corr)| > 0.5) and targeted bioactive metabolites (*p* < 0.05), followed by filtering based on fold change (|fold change| > 10) between PS and YG. Bioactive metabolites are denoted by the letter (a) and bold, and detailed information is available in Table S4.

A statistical approach was used to investigate the marker metabolites that contribute to the differentiation between PS and YG. The discriminant metabolites selected from the untargeted analysis (VIP > 1.0, *p* < 0.05) were further filtered using |p(corr)| > 0.5. Bioactive metabolites identified through targeted analysis, showing significant differences between PS and YG (*p* < 0.05), were integrated to complement the untargeted candidates. Finally, applying a |fold change| > 10 threshold led to the identification of 27 PS-specific-and 17 YG-specific marker metabolites (Figure 3).

PS-specific markers included GABA; 11 amino acids (glutamic acid, threonine, isoleucine, histidine, phenylalanine, tryptophan, aspartic acid, methionine, lysine, serine, and arginine), *N*-acetylphenylalanine, *N*-acetylleucine, 6 *γ*-glutamylpeptides (*γ*-glutamylleucine, *γ*-glutamylmethionine, *γ*-glutamylphenylalanine, *γ*-glutamylvaline, *γ*-glutamylcysteine, and *γ*-glutamyltryptophan), indole-3-acrylic acid, indole-3-propionic acid, indole-3-acetic acid, phenylethylamine, ascorbic acid, nicotinic acid, and niacinamide. Amino acids serve as fundamental substrates in cellular metabolism, and essential amino acids must be supplied through diet. Several amino acids, including glutamic acid, tryptophan, aspartic acid, methionine, and arginine, play crucial roles in physiological regulation [39].

By contrast, the YG-specific markers included creatine, carnitine, and acylcarnitines (propionylcarnitine, butyrylcarnitine, and valerylcarnitine), three carbohydrates (disaccharide phosphate, *N*-acetylhexosamine, and *N*-acetylhexosamine phosphate), amino valeric acid betaine, phenylacetylglycine, orotic acid, uric acid, hippuric acid, glycocholic acid, taurocholic acid, indole-3-carboxylic acid-*O*-sulfate, and riboflavin. Previous studies have reported that improvements in constipation following the consumption of fermented milk products are significantly and positively correlated with changes in serum acylcarnitine levels [40]. This observation indicates that the acylcarnitines identified as YG-specific markers in the present study may reflect the metabolic characteristics associated with YG consumption. These markers provide a foundation for exploring the potential functional differences between PS and YG and may serve as candidate metabolites for future studies investigating health-related effects.

### 2.4. Antioxidant and Anti-Glycation Activity of Probiotic Supplements and Yogurt

**Figure 4:**
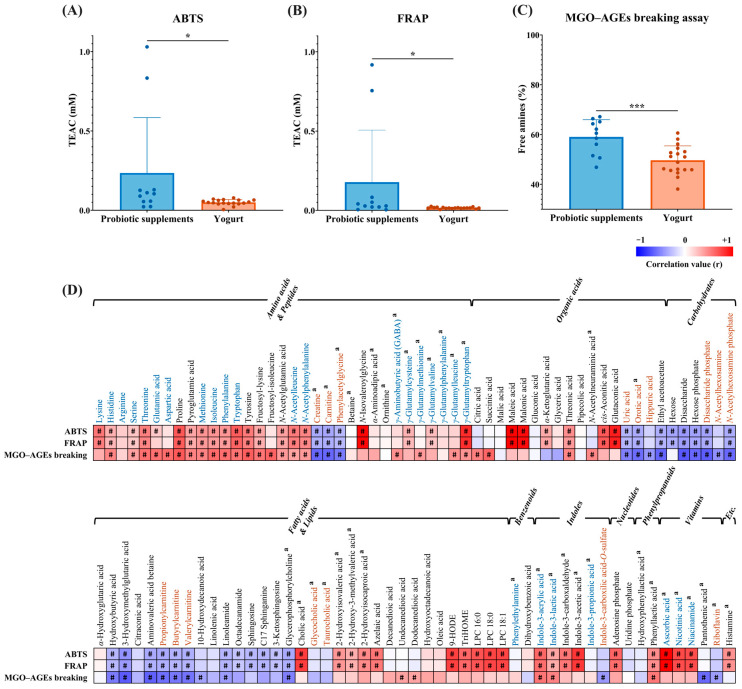
Bioactivity assays and correlation analysis in probiotic supplements (PS) and yogurt (YG). (**A**) ABTS radical scavenging assay; (**B**) ferric reducing antioxidant power (FRAP) assay; (**C**) MGO–AGE breaking assay. The *y*-axis in (**A**,**B**) represents Trolox equivalent antioxidant capacity (TEAC), while the *y*-axis in (**C**) represents the level of free amines released during MGO–AGE breaking. (**D**) Pearson’s correlation map between the relative abundance of metabolites and bioactivities in PS and YG. PS- and YG-specific markers determined in Figure 3 are shown in blue and orange text, respectively. Bioactive metabolites are marked with the letter (a), and detailed information is provided in Table S4. Bioactivity assay significance is indicated by asterisks (* *p* < 0.05, *** *p* < 0.001), while significant correlations are marked with # (*p* < 0.05).

To assess the functional relevance of metabolite differences between PS and YG, antioxidant and anti-glycation activities were evaluated using ABTS, FRAP, and MGO–AGE breaking assays. The ABTS and FRAP results were expressed as TEAC, and the anti-glycation activity was quantified based on the release of free amines during MGO–AGE breaking. PS exhibited significantly higher antioxidant activities than YG in both ABTS and FRAP assays (Figure 4A,B). Two PS samples showed relatively high antioxidant activity (TEAC > 0.7). To determine whether these high values substantially affected the group-level comparison, additional analyses were performed excluding the two samples. As shown in Figure S2A,B, the statistically significant difference between PS and YG remained consistent. In addition, PS showed a significantly higher anti-glycation activity in the MGO–AGE breaking assay than in the YG assay (Figure 4C).

To explore the metabolites potentially contributing to these bioactivities, a Pearson correlation analysis was performed between the metabolites and antioxidant and anti-glycation activities (Figure 4D). As a result, 26 metabolites showed significant positive correlations with antioxidant activity, and 14 metabolites showed significant positive correlations with anti-glycation activity (r > 0.5, *p* < 0.05). Metabolites positively correlated with antioxidant activity included free amino acids (lysine, threonine, proline, tryptophan, and tyrosine), *N*-acetylleucine, *N*-isovaleroylglycine, *γ*-glutamyltryptophan, organic acids (maleic acid, malonic acid, *cis*-aconitic acid, and glutaconic acid), cholic acid, azelaic acid, oxylipins (9-HODE and TriHOME), lysophospholipids (LPC 16:0, LPC 18:0, and LPC 18:1), indole derivatives (indole-3-acrylic acid, indole-3-carboxaldehyde, and indole-3-acetic acid), adenosine phosphate, ascorbic acid, nicotinic acid, and nicotinamide. Previous studies have reported that several amino acids, including proline, tryptophan, and tyrosine, exhibit free radical-scavenging activity [41,42]. In addition, indole-3-acrylic acid, indole-3-carboxaldehyde, and indole-3-acetic acid, which have been reported as bioactive metabolites, are associated with antioxidant effects [43,44,45]. Similarly, the anti-glycation activity was significantly positively correlated with amino acids (threonine, glutamic acid, aspartic acid, proline, isoleucine, phenylalanine, tryptophan, and tyrosine), fructosyl-lysine, fructosyl-isoleucine, citric acid, succinic acid, indole-3-acrylic acid, and phenyllactic acid. The anti-glycation effects of essential amino acids, including isoleucine, phenylalanine, tryptophan, and tyrosine, have been reported previously [46].

**Figure 5:**
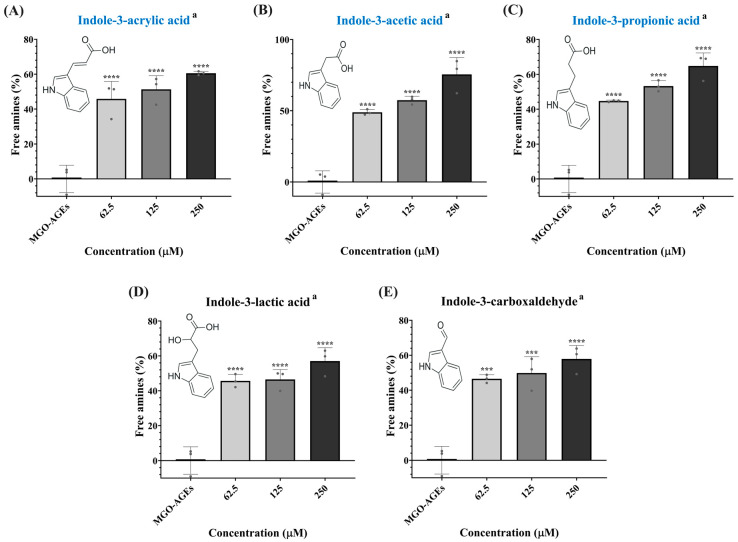
Anti-glycation activity of indole derivatives identified in probiotic supplements and yogurt from MGO–AGEs breaking assay. (**A**) Indole-3-acrylic acid; (**B**) indole-3-acetic acid; (**C**) indole-3-propionic acid; (**D**) indole-3-lactic acid; and (**E**) indole-3-carboxadehyde. PS-specific marker metabolites determined in Figure 3 are shown in blue text. Bioactive metabolites are denoted by the letter (a), and detailed information is available in Table S4. Results are presented as mean ± SD of technical replicates (*n* = 3). *** *p* < 0.001 and **** *p* < 0.0001 vs. MGO–AGEs (control).

Interestingly, indole-3-acrylic acid was identified as a candidate metabolite correlated with both antioxidant and anti-glycation activities, and was also determined to be a PS-specific marker. Indole-3-acrylic acid—a tryptophan-derived microbial metabolite—has been reported to exert various physiological benefits, including anti-inflammatory effects, antioxidant activity, enhancement of intestinal barrier function, and immunomodulatory properties [29,45]. Microbial indole metabolites have attracted considerable interest as key mediators of host–microbe interactions [47]; however, their anti-glycation activities remain incompletely understood. In this context, considering the relatively higher abundance of indole derivatives in PS than in YG (Figure 2), the anti-glycation activity was evaluated beyond indole-3-acrylic acid across indole derivatives as a chemical class. The anti-glycation activity was assessed using the MGO–AGE breaking assay for 12 metabolites, including tryptophan and five indole derivatives detected in PS and YG (indole-3-acrylic acid, indole-3-acetic acid, indole-3-propionic acid, indole-3-lactic acid, and indole-3-carboxaldehyde), as well as six additional metabolites from the microbial tryptophan–indole pathway (indole, indole-3-acetamide, indole-3-acetaldehyde, indoxyl-3-sulfate, indole-3-pyruvic acid, and tryptamine). Among these, indole-3-acrylic acid, indole-3-acetic acid, indole-3-propionic acid, indole-3-lactic acid, indole-3-carboxaldehyde, indole, indole-3-acetamide, indole-3-pyruvic acid, and tryptamine exhibited significant AGE-breaking activity compared with the control (Figure 5 and Figure S3). By contrast, tryptophan, indole-3-acetaldehyde, and indoxyl-3-sulfate did not exhibit significant AGE-breaking activity. At 250 μM, the relative anti-glycation activities followed the order indole-3-acetic acid (76%) > indole-3-pyruvic acid (72%) > tryptamine (66%) > indole-3-propionic acid (65%) > indole-3-acrylic acid (61%) > indole-3-carboxaldehyde (58%) > indole-3-lactic acid (57%) > indole-3-acetamide (51%) > indole (50%). Notably, indole-3-acrylic acid, indole-3-acetic acid, and indole-3-propionic acid, which were identified as PS-specific marker metabolites, exhibited relatively high anti-glycation activities (>60%) (Figure 5A–C). Previous studies have reported that tryptophan and indole can inhibit AGE formation through dicarbonyl trapping mechanisms, and such anti-glycation effects have been attributed to their structural features, particularly the indole ring. The indole scaffold is known to undergo electrophilic aromatic substitution reactions, suggesting a potential capacity to interact with reactive 1,2-dicarbonyl compounds [48,49]. By contrast, the anti-glycation activity observed in the AGE-breaking assay varied among the indole compounds and was inconsistently associated with the presence of an indole ring. This pattern indicates that the differences in AGE-breaking activity among the indole compounds may be influenced by variations in the side-chain structures rather than by the indole ring itself. Taken together, these results suggest that the relatively high anti-glycation activity observed in the PS may be partly associated with the high abundance of indole derivatives. These compounds are known to arise from microbial tryptophan metabolism in probiotic strains, and it is plausible that a portion of the indole derivatives detected in PS accumulated during strain cultivation and were retained through downstream processing [17]. However, as all samples analyzed in this study were obtained from the Korean market, potential regional variations in product composition cannot be excluded. Furthermore, the indole validation results presented here are based on in vitro assays; therefore, digestion, bioavailability, and in vivo relevance should be carefully considered in future studies. Nevertheless, the comparative metabolomic approach applied in this study provides valuable insight into compositional and functional differences between PS and YG. The identified marker metabolites represent potential bioactive candidates that may contribute to the functional characteristics distinguishing PS and YG.

## 3. Materials and Methods

### 3.1. Chemicals and Reagents

HPLC-grade water, methanol, and acetonitrile were purchased from Thermo Fisher Scientific (Waltham, MA, USA). Acetic acid, formic acid, 3-nitrophenylhydrazine hydrochloride (3-NPH), *N*-(3-dimethylaminopropyl)-*N*-ethyl carbodiimide hydrochloride (EDC), 6-hydroxy-2,5,7,8-tetramethylchroman-2-carboxylic acid (Trolox), 2,2′-azinobis (3-ethylbenzothiazoline-6-sulfonic acid) diammonium salt (ABTS), potassium persulfate, hydrochloride, iron(III) chloride hexahydrate, 2,4,6-tris(2-pyridyl)-s-triazine (TPTZ), MGO, bovine serum albumin (BSA), phosphate-buffered saline (PBS), sodium azide, 2,4,6-trinitrobenzene sulfonic acid, sodium bicarbonate, and all standard compounds were obtained from Sigma-Aldrich (St. Louis, MO, USA). Sodium dodecyl sulfate (SDS) was purchased from Amresco (Solon, OH, USA).

### 3.2. Sample Collection

A total of 29 commercially available probiotic products—11 PS and 18 YG—were purchased from Korean online retail platforms and official brand websites. The ingredients and microbial information of all products are provided in Table S1. YG products were delivered in insulated packages with ice packs and stored at −20 °C upon arrival until metabolite extraction, whereas PS products were stored at room temperature.

### 3.3. Untargeted Metabolomics Analysis

#### 3.3.1. Sample Preparation

PS samples (50 mg) were suspended in 1 mL of extraction solvent (AMW), consisting of 0.4 mL of acetonitrile, 0.4 mL of methanol, and 0.2 mL of water containing an internal standard solution (2-chloro-L-phenylalanine, 10 µg/mL in AMW). The resuspended mixtures were vortexed for 30 s, sonicated for 30 min, and centrifuged at 12,000 rpm for 10 min at 4 °C. The supernatant was filtered using 0.2 µm polytetrafluoroethylene (PTFE) syringe filters (Chromdisc, Daegu, Republic of Korea).

YG samples (50 mg) were suspended in 1 mL AMW. The resuspended mixtures were vortexed for 30 s, sonicated for 10 min, and left to stand at 4 °C for 1 h. The mixture was centrifuged at 12,000 rpm for 15 min at 4 °C. The supernatant was filtered using 0.2 µm PTFE syringe filters.

An 800 µL aliquot of the filtered supernatants from each PS and YG sample was dried using a speed vacuum concentrator. Three independent commercial products were prepared and analyzed as biological replicates. Quality control (QC) samples were prepared by pooling equal volumes of the individual extracts. All extracts were stored at −20 °C until the untargeted metabolite and bioactivity analyses were performed.

#### 3.3.2. Ultrahigh-Performance Liquid Chromatography–Orbitrap-Tandem Mass Spectrometry (UHPLC–Orbitrap-MS/MS) Analysis

The dried extract was reconstituted in AMW (2.5 mg/mL) and filtered using a 0.2 µm PTFE filter for analysis using UHPLC–Orbitrap-MS/MS. The UHPLC system was equipped with a Vanquish Binary Pump C (Thermo Fisher Scientific) coupled with an autosampler and column compartment. Chromatographic separation was performed using a Waters ACQUITY UPLC HSS T3 column (100 mm × 2.1 mm, 1.8 μm particle size; Waters, Wexford, Ireland) and a mobile phase consisting of 0.1% (*v*/*v*) formic acid in water (Solvent A) and 0.1% (*v*/*v*) formic acid in acetonitrile (Solvent B). The following gradient condition was employed: 0–1 min, 5% B; 1–10 min, 5%–100% B; 10–11 min, 100% B; 11–13 min, 100%–5% B; and 13–15 min, 5% B. The flow rate, injection volume, and column temperature were 0.3 mL/min, 5 μL, and 40 °C, respectively. Mass spectra were recorded in the range of 100–1500 *m*/*z* using an Orbitrap Exploris 120 Mass Spectrometer (Orbitrap MS, Thermo Fisher Scientific) coupled with an HESI-II (H-ESI) probe. The probe heater and capillary temperatures were set to 300 and 320 °C, respectively. The capillary voltage was set to 3.5 kV in the positive mode and 2.8 kV in the negative mode. Each sample was injected in a randomized order to minimize systematic bias. Blank solvent washes were performed every 10 injections, and QC samples were analyzed every 20 injections.

### 3.4. Targeted Metabolomics Analysis

#### 3.4.1. Sample Preparation for Short-Chain Fatty Acid Analysis

SCFA analyses were performed using the 3-NPH derivatization method [16] with some modifications. To evaluate the SCFA differences between the two product types, pooled raw product samples were prepared by combining equal weights of individual PS and YG products. For analysis, 1 g of each pooled PS and pooled YG sample was mixed with 1 mL of water and homogenized using a bead-beating step (MM400 mixer mill, Retsch^®^, Haan, Germany) at a frequency of 30 s^−1^ for 10 min. The mixture was centrifuged at 12,000 rpm for 10 min, and the supernatant was collected for derivatization. For derivatization, 50 µL of the supernatant from the pooled sample was mixed with 20 µL of 0.2 M 3-NPH solution and 20 µL of 0.12 mM EDC solution containing 6% pyridine. The mixture was incubated at 40 °C for 30 min, and then 200 µL of 1% formic acid was added to quench the reaction. The final solution was filtered through a 0.2 µm PTFE syringe filter prior to UHPLC–triple quadrupole tandem mass spectrometry analysis.

#### 3.4.2. UHPLC–Triple Quadrupole Tandem Mass Spectrometry (UHPLC–Triple Q-MS/MS) Analysis

The analysis was performed using a Shimadzu LC-30AD system (Shimadzu Corp., Kyoto, Japan) coupled with a triple quadrupole tandem mass spectrometer and an electrospray source (LCMS-8040, Shimadzu Corp.). Chromatographic separation was conducted using an Acquity UPLC BEH C18 column (100 mm × 2.1 mm × 1.7 μm particle size; Waters Corp., Milford, MA, USA). The mobile phases consisted of water with 1% formic acid (A) and acetonitrile with 1% formic acid (B). The following gradient condition was employed: 0–2 min, 15% B; 2–25 min, 15%–40% B; 25–26.5 min, 100% B; 26.5–27 min, 100%–15% B; and 27–28 min, 15% B. The flow rate, injection volume, and column temperature were 0.35 mL/min, 10 μL, and 30 °C, respectively. Target analytes were monitored using the scheduled multiple reaction monitoring mode with mass transitions (Table S2). After the analysis, each peak area was quantified using the calibration curve of the corresponding target compound, and the values were subsequently normalized to the extraction yield to allow comparison at an equivalent extract concentration. All analyses were performed in triplicate.

### 3.5. Bioactivity Assay Analysis

#### 3.5.1. Antioxidant Activity

Antioxidant activity was evaluated using the ABTS radical scavenging and ferric reducing antioxidant power (FRAP) assays. The protocols were adapted from previously reported methods [50], with slight modifications. For the ABTS assay, a 7 mM ABTS stock solution was prepared by dissolving ABTS in 2.45 mM potassium persulfate and incubating the mixture in a water bath at 60 °C for 20 min. The solution was then stored at 4 °C overnight. Before analysis, the ABTS stock solution was diluted with water and adjusted to an absorbance of 0.70 ± 0.02 at 750 nm. Subsequently, 10 µL of the sample extract (5 mg/mL) or compounds was added to 190 µL of the diluted ABTS solution in a 96-well plate and incubated for 7 min at room temperature in the dark. The absorbance was measured at 750 nm using a spectrophotometer (Spectronic Genesys 6; Thermo Electron, Madison, WI, USA). The FRAP assay was performed by combining an acetate buffer (300 mM, pH 3.6), TPTZ (10 mM in 40 mM HCl), and FeCl_3_ (20 mM) in a 10:1:1 (*v*/*v*/*v*) ratio to prepare the FRAP reagent. A 10 µL aliquot of the sample extract (5 mg/mL) or compounds was mixed with 300 µL of the FRAP reagent in a 96-well plate and incubated at room temperature for 6 min in the dark. The absorbance was measured at 570 nm. All antioxidant assays were performed in triplicate, and the results are expressed as Trolox equivalent concentrations (TEAC). Standard curves were generated from 0.015625–1 mM concentration.

#### 3.5.2. AGE-Breaking Activity

The AGE-breaking activity assay was performed according to Subedi et al. [51], with some modifications. MGO-modified BSA (MGO-BSA) was produced by incubating 5 mg/mL BSA and 10 mM MGO in PBS in the presence of 0.02% sodium azide (pH 7.4) at 37 °C for 7 d. After incubation, the resultant AGEs were dialyzed using a 7 kDa molecular-weight cut-off membrane. The filtered solution was evaporated and then freeze dried, yielding the AGE powders. Then, 100 µL of MGO-BSA solution (1 mg/mL) was mixed with 100 µL of the sample extracts (0.2 mg/mL) or compounds and then incubated for 24 h. After incubation, 200 µL of 4% NaHCO_3_ (pH 8.5) and 200 µL of 0.1% TNBS were added, and the solution was incubated at 37 °C for 2 h. After the reaction, 100 µL of 0.1% SDS and 100 µL of 1 N HCl were added. AGE-breaking activity was measured at 340 nm using a spectrophotometer. All experiments were performed in triplicates, and the mixture without MGO was used as the blank solution.

### 3.6. Data Processing and Statistical Analysis

The UHPLC–Orbitrap-MS/MS raw data were acquired and preprocessed using Thermo Freestyle software (version 1.8.63.0; Thermo Fisher Scientific). Peak picking was performed using ProteoWizard software (version 3.0.24214), and the data were output in mzXML format (*.mzXML) before being converted to ABF format (*.abf) using ABF Converter software (version 1.3.8550.28994; https://www.reifycs.com/abfconverter/, accessed on 20 February 2026). After conversion, retention time correction, alignment, and blank subtraction were performed using the MS-DIAL software (version 5.4.241004). Blank subtraction was performed to minimize the influence of solvent-derived peaks and background noise. Multivariate statistical analyses, including principal component analysis (PCA) and orthogonal partial least squares discriminant analysis (OPLS-DA), were performed using SIMCA-P+ software (version 15.0.2; Umetrics, Umea, Sweden). Significantly discriminant metabolites were selected based on a Variable Importance in Projection (VIP) value greater than 1.0 and significant differences (raw *p* < 0.05), which were assessed by independent sample *t*-tests using STATISTICA 7 (StatSoft Inc., Tulsa, OK, USA). To control for multiple testing, *p*-values were further adjusted using the Benjamini–Hochberg false discovery rate (FDR) method, and the adjusted values are provided in Table S3. Metabolites were identified according to the Metabolite Standards Initiative (MSI) criteria [52]. MSI Level 1 identification requires at least two orthogonal criteria—accurate mass, retention time, and MS/MS fragmentation—matched to an in-house library of authentic standards analyzed under identical conditions [53]. MSI Level 2 compounds were tentatively identified based on similarities in MS/MS spectra by referencing publicly available databases, including MassBank of North America (MoNA, https://massbank.us/), PubChem (https://pubchem.ncbi.nlm.nih.gov/), and the Human Metabolome Database (HMDB, https://hmdb.ca/). Pearson’s correlation coefficients were calculated using PASW Statistics 18 software (SPSS Inc., Chicago, IL, USA). Volcano plots were generated using MetaboAnalyst 6.0, a web-based platform (https://www.metaboanalyst.ca/), and visualized using GraphPad Prism software (version 10.4.1; La Jolla, CA, USA; http://www.graphpad.com).

## 4. Conclusions

This study applied integrated untargeted and targeted metabolomics approaches to compare the metabolite profiles of PS and YG, which represent two distinct probiotic product formats, and explored their potential functional relevance. PS were relatively enriched in amino acids and bioactive metabolites, including GABA, *γ*-glutamylpeptides, and indole derivatives, which were identified as PS-specific marker metabolites. By contrast, YG exhibited a higher relative abundance of carbohydrates, acylcarnitines, identified as YG-specific markers metabolites. In addition, PS exhibited significantly higher antioxidant and anti-glycation activities than YG in vitro. Notably, indole-3-acrylic acid, indole-3-acetic acid, and indole-3-propionic acid have been identified as PS-specific bioactive marker metabolites that exhibit anti-glycation activity. These findings indicate that the metabolomic differences between PS and YG are associated with differences in their functional properties. Accordingly, the marker metabolites identified in this study may serve as potential bioactive candidates for future studies investigating their potential health effects and as metabolomic indicators for the product characterization of PS and YG.

## Data Availability

The data presented in this study are available from the corresponding author upon reasonable request.

## Supplementary Materials

The supporting information can be downloaded at: https://www.mdpi.com/article/10.3390/ijms27052180/s1. References [9,25,27,28,29,30,31,33,35,45,54,55,56,57,58,59,60,61,62,63,64,65,66,67,68,69,70,71,72,73,74,75,76,77,78,79,80,81,82,83,84,85] are cited in the supplementary materials (Table S4).
