# Supplementary material for: Integrated Untargeted and Targeted Metabolomics Reveals Distinct Bioactive Metabolite Profiles Between Probiotic Supplements and Yogurt
Source: Int J Mol Sci. 2026 Feb 26;27(5):2180. doi: 10.3390/ijms27052180 (PMC12984118; doi:10.3390/ijms27052180)
Supplement: Supplementary file 1 [file ijms-27-02180-s001.zip › Supplementary material.pdf]

Supplementary material

Integrated untargeted and targeted metabolomics reveals distinct bioactive metabolite profiles between probiotic supplements and yogurt

Sang Hyeon Noh, Su-Hyun Kim, Do Hoon Kwon, and Choong Hwan Lee*

Department of Bioscience and Biotechnology, Konkuk University, Seoul, Republic of Korea

*Corresponding author:

Email: chlee123@konkuk.ac.kr (C.H.L.)

Tables

Table S1. Sample information on probiotic supplements and yogurt purchased from Korean online retail platforms and official brand websites.

Table S2. MRM transition for targeted analysis of short-chain fatty acids based on UHPLC–Triple Q-MS/MS.

Table S3. Identification metabolites in probiotic supplements and yogurt based on UHPLC–Orbitrap-MS/MS.

Table S4. List of bioactive metabolites associated with probiotics or yogurt.

Figures

Fig. S1. Principal component analysis (PCA) score plots in integrated positive and negative ionization modes between probiotic supplements (blue circles), yogurt (orange circles) and quality control samples (black circles) based on UHPLC–Orbitrap-MS/MS.

Fig. S2. Antioxidant assays after exclusion of two samples with extreme ABTS and FRAP values in probiotic supplements (PS) and yogurt (YG). (A) ABTS radical scavenging assay; (B) ferric reducing antioxidant power (FRAP) assay. The y-axis in (A) and (B) represents Trolox equivalent antioxidant capacity (TEAC). Statistical significance is indicated by asterisks (*p < 0.05).

Fig. S3. Antiglycation activity of tryptophan and indole derivatives in the MGO–AGEs breaking assay. (A) Tryptophan; (B) indole; (C) indole-3-pyruvic acid; (D) tryptamine; (E) indole-3-acetamide; (F) indole-3-acetaldehyde; and (G) indoxyl-3-sulfate. PS-specific marker metabolites determined in Fig. 3 are shown in blue text. Bioactive metabolites are denoted by the letter (a), and detailed information is available in Table S4. Results are presented as mean ± SD of technical replicates (n = 3). ****p < 0.0001 vs. MGO–AGEs (control); ns indicates not significant (p > 0.05).

Table S1. Sample information on probiotic supplements and yogurt purchased from Korean online retail platforms and official brand websites.

Product	Sample number	Ingredients [a]	Microbial Species [a]
Probiotics Supplements	PS1	Rice flour, Maltodextrin	*Lactobacillus acidophilus*
	PS2	Microcrystalline cellulose (plant fiber), Sillica, Magnesium stearate	*Lactobacillus gasseri*
	PS3	Heat-killed Bifidobacterium breve culture powder, glucose syrup solids, maltodextrin, fructooligosaccharide, magnesium stearate	*Bifidobacterium breve*
	PS4	Mixed lactose [lactose (USA), dextrin], indigestible maltodextrin, galactooligosaccharide, inulin/chicory extract (Belgium), fructooligosaccharide, silicon dioxide, magnesium stearate, beta-cyclodextrin	*Lactobacillus gasseri*
	PS5	Maltodextrin, microcrystalline cellulose, magnesium stearate	*Bifidobacterium animalis subsp. lactis* (BB-12), *Lacticaseibacillus rhamnosus* (LGG)
	PS6	Microcrystalline cellulose, vegetarian magnesium stearate, silicon dioxide	*Lactobacillus acidophilus, Bifidobacterium lactis, Bifidobacterium longum, Lactobacillus rhamnosus, Bifidobacterium breve, Lactobacillus casei, Lactobacillus salivarius, Lactobacillus plantarum*
	PS7	Microcrystalline cellulose, Stearic acid (vegetable source), Silicon Dioxide	*Lactobacillus acidophilus, Bifidobacterium lactis, Bifidobacterium longum*
	PS8	Chicory root extract powder, fructooligosaccharide, oat dietary fiber, HK shiitake mushroom heat-killed cells, dried yeast (*Saccharomyces cerevisiae*), bamboo sap extract powder	*Lacticaseibacillus rhamnosus* (LGG)*, Lacticaseibacillus paracasei, Lactiplantibacillus plantarum, Lactobacillus acidophilus, Bifidobacterium animalis subsp. lactis, Bifidobacterium bifidum*
	PS9	Indigestible maltodextrin, 15-grain fermented enzyme powder [15-grain blend powder (brown rice, whole wheat, barley, buckwheat, potato, soybean, sorghum, Job's tears, corn, rye, black bean, millet, red bean, sweet potato, oat), Bacillus amyloliquefaciens], fructooligosaccharide, inulin/chicory extract, rice fermented powder, cottonseed oil powder, probiotic culture powder	*Lactiplantibacillus plantarum, Lactobacillus acidophilus, Streptococcus thermophilus, Lactococcus lactis, Bifidobacterium animalis subsp. lactis, Bifidobacterium bifidum*
	PS10	Indigestible maltodextrin, corn starch, maltodextrin, alpha corn starch, fructooligosaccharide, potassium phytate, vitamin C, isolated soy protein, hydrated hyaluronic acid, guar gum, trehalose, dried yeast (selenium-containing Canada origin), zinc oxide, microcrystalline cellulose, apple dietary fiber (USA origin), oat dietary fiber (Germany origin), dietary fiber (fenugreek seed extract), Bacillus coagulans, galactooligosaccharide, magnesium stearate, silicon dioxide, propolis extract powder, HK shiitake mushroom mycelium, L-arginine, reishi mushroom fruiting body extract	*Lacticaseibacillus rhamnosus, Lactiplantibacillus plantarum, Enterococcus faecium, Bifidobacterium animalis subsp. lactis, Bifidobacterium longum*
	PS11	Corn starch, maltodextrin, granulated sugar, isolated soy protein, guar gum, hyaluronic acid, microcrystalline cellulose, zinc oxide, probiotic blend powder, mixed lactose (lactose, dextrin), magnesium stearate, fructooligosaccharide, probiotic culture powder, anhydrous crystalline glucose	*Enterococcus faecium, Lactiplantibacillus plantarum, Lactobacillus acidophilus, Lacticaseibacillus casei, Lacticaseibacillus rhamnosus, Streptococcus thermophilus, Ligilactobacillus salivarius, Bifidobacterium animalis subsp. Lactis, Bifidobacterium bifidum, Bifidobacterium breve, Bifidobacterium longum, Lactobacillus delbrueckii subsp. bulgaricus, Lactobacillus gasseri, Lacticaseibacillus paracasei, Lactococcus lactis*
Yogurt	YG1	Raw milk (Korea) 99.996%, Lactic acid bacteria mix powder (Denmark)	Lactic acid bacteria [b]
	YG2	Raw milk 99.96% (Korean), Mixed lactic acid bacteria 0.04%	Lactic acid bacteria [b]
	YG3	Raw Milk 99.8% (Korean), Lactic acid bacteria 0.2% (Italy)	Lactic acid bacteria [b]
	YG4	Raw milk 99.996% (Korean), Lactic acid bacteria 0.004%	Lactic acid bacteria [b]
	YG5	Raw milk 99.996% (Korean), Lactic acid bacteria 0.004%	Lactic acid bacteria [b]
	YG6	Raw Milk 99.9% (Korean), Lactic acid bacteria 0.1% (Denmark)	Lactic acid bacteria [b]
	YG7	Raw Milk 99.99% (Korean), Lactic acid bacteria 0.01%	Lactic acid bacteria [b]

YG8	Raw milk 99.996% (Korea), Lactic acid bacteria 0.004%	Lactic acid bacteria [b]
YG9	Raw milk (99.996%, Korean), Lactic acid bacteria 0.004%	Lactic acid bacteria [b]
YG10	Raw milk 99.99% (Korean), Lactic acid bacteria 4 billion or more per 400g	*Lactobacillus acidophilus, Bifidobacterium longum, Streptococcus thermophilus*
YG11	Raw milk 99.99% (Korean), Lactic acid bacteria	Lactic acid bacteria [b]
YG12	Raw milk (Korea) 97%, Mixed skim milk powder (Netherlands), Whey protein powder (Austria), Fish gelatin, Emulsifier, Lactic acid bacteria 5 billion or more/g	*Streptococcus thermophilus, Lactobacillus delbrueckii subsp. bulgaricus, Lactobacillus rhamnosus* (LGG)
YG13	Raw milk 98.5% (Korean), Mixed formulation (modified starch, dextrin), Lactic acid bacteria strain (240 billion or more/430g)	Lactic acid bacteria [b]
YG14	Raw milk (Korean) 98.04%, Skim milk powder (Korean) 0.73%, Whey protein isolate (Denmark), Gelatin (fish), Modified starch, Amide pectin, Lactic acid bacteria	Lactic acid bacteria [b]
YG15	Raw milk (Korean), Purified water, Fructo-oligosaccharides, Skim milk powder (Netherlands), Crystalline fructose, Mixed milk powder (Netherlands), Modified starch, Maltodextrin, Concentrated milk protein, Amide pectin, Probiotics, Stevia enzyme-treated, Zinc oxide	Probiotics [b]
YG16	Raw milk 84.9888% (Korean), Concentrated milk 15% (Korean), LB-9 probiotic mix powder 0.0037%, Lactic acid bacteria 4 billion CFU/400g	Probiotics [b], Lactic acid bacteria [b]
YG17	Raw milk (Korean, Grade 1A bacteria count) 87.74%, Purified water, Whey protein isolate (Denmark), Modified starch, Gelatin, Amide pectin, Lactic acid bacteria (Denmark)	Lactic acid bacteria [b]
YG18	Raw milk (Korean), Purified water, Sweetis DS400, Mixed skim milk powder (Netherlands), Y-Cream (Korean), Crystalline fructose, Modified starch, fructose, Milk protein powder, Milk concentrate powder, Emulsifier, Guar gum, Amide pectin, Synthetic flavor, Microbiome synergy complex material, Lactase enzyme, Bifidobacterium (BB-12) 100,000 CFU or more/g, Mixed lactic acid bacteria 5 billion or more per cup	*Bifidobacterium animalis subsp. lactis* (BB-12), Lactic acid bacteria [b]

[a] Ingredients and Microbial species information was obtained from the product label

[b] Species information not provided

Table S2. MRM transition for targeted analysis of short-chain fatty acids based on UHPLC–Triple Q-MS/MS.

No.	Metabolite	Ion mode	RT (min) [a]	Precursor ion (m/z)	Product ion (m/z)
Short-chain fatty acids					
1	Acetic acid	Negative	5.01	194.10	152.10, 137.05
2	Propionic acid	Negative	7.77	208.10	137.00, 152.00, 162.05
3	Butyric acid	Negative	11.83	222.10	137.05, 152.05, 179.10

[a] RT, Retention time

Table S3. Identification metabolites in probiotic supplements and yogurt based on UHPLC–Orbitrap-MS/MS.

No.	RT (min) [a]	Tentative Identification	Comparison of Probiotics Supplement and Yogurt						Measured mass (m/z)		M.W. [c]	MS/MS fragments	Molecular formula	Delta ppm	MSI level	Identification
			VIP [b]	p[1]	p(corr)	log2(FC)	*p*-value	FDR (Benjamini–Hochberg)	[M-H]-	[M+H]+						
Amino acids & Peptides																
1	0.66	Ornithine	1.17	0.00	-0.45	3.23	7.00E-06	9.94E-06		132.0899	133.0971	(+)116, 70, 133, 115, 123	$C_5H_{12}N_2O_2$	0.82	2	MoNA
2	0.66	Lysine	5.66	-0.02	-0.76	3.81	1.00E-17	3.28E-17	-	147.1126	146.1055	(+)130, 84, 147, 129, 72	$C_6H_{14}N_2O_2$	-1.53	1	In-House Library
3	0.66	Fructosyl-lysine	1.68	-0.01	-0.55	1.61	1.00E-07	2.33E-07	-	309.165	308.1584	(+)225, 128, 273, 84, 130	$C_{12}H_{24}N_2O_7$	-1.9	2	MoNA
4	0.67	Histidine	3.87	-0.01	-0.88	3.80	1.00E-28	6.29E-28	-	156.0765	155.0695	(+)110, 156, 130, 95, 130	$C_6H_9N_3O_2$	-1.48	2	MoNA
5	0.67	Arginine	5.23	-0.02	-0.52	3.52	1.00E-06	1.52E-06	-	175.1186	174.1117	(+)175, 60, 116, 70, 158	$C_6H_{14}N_4O_2$	-1.8	1	In-House Library
6	0.74	Serine	2.47	-0.01	-0.75	4.20	1.00E-16	3.32E-16	-	106.0496	105.0426	(+)60, 106, 88, 105, 70	$C_3H_7NO_3$	1.56	1	In-House Library
7	0.74	Carnitine	10.62	0.04	0.97	-5.28	1.00E-61	1.00E-59	-	162.1125	161.1052	(+)162, 103, 60, 163, 102	$C_7H_{15}NO_3$	-2.9	2	MoNA
8	0.75	Aspartic acid	3.85	-0.01	-0.83	4.26	2.00E-22	9.53E-22	-	134.0446	133.0375	(+)88, 74, 134, 116, 46	$C_4H_7NO_4$	1.6	1	In-House Library
9	0.76	Threonine	6.63	-0.02	-0.91	4.26	4.00E-32	3.82E-31	-	120.0653	119.0582	(+)74, 102, 120, 56, 84	$C_4H_9NO_3$	2.1	2	MoNA
10	0.76	Creatine	18.47	0.06	0.98	-6.84	9.00E-64	1.28E-61	-	132.0764	131.0695	(+)132, 90, 44, 133, 85	$C_4H_9N_3O_2$	-3.01	2	MoNA
11	0.76	Glutamic acid	17.20	-0.06	-0.91	3.59	1.00E-32	1.21E-31	146.046	148.06	147.0532	(+)84, 130, 102, 148, 147	$C_5H_9NO_4$	-2.64	1	In-House Library
12	0.77	Betaine	18.99	-0.06	-0.60	2.62	1.00E-10	2.55E-10	-	118.0859	117.079	(+)118, 59, 69, 119, 117	$C_5H_{11}NO_2$	-2.69	2	MoNA
13	0.79	Proline	12.11	-0.04	-0.80	1.41	4.00E-18	1.44E-17	-	116.0704	115.0633	(+)116, 70, 117, 69, 71	$C_5H_9NO_2$	-2.06	2	MoNA
14	0.8	γ-Aminobutyric acid (GABA)	3.39	-0.01	-0.65	3.88	1.00E-11	2.62E-11	-	104.0707	103.0634	(+)87, 104, 86, 69, 43	$C_4H_9NO_2$	-1.57	2	Pubchem
15	0.82	Fructosyl-isoleucine	11.74	-0.04	-0.60	3.10	2.00E-08	3.84E-08	-	294.1541	293.1475	(+)276, 258, 230, 210, 132	$C_{12}H_{23}NO_7$	-2.09	2	MoNA
16	0.83	Methionine	6.57	-0.02	-0.80	4.30	1.00E-18	3.42E-18	-	150.058	149.051	(+)104, 133, 56, 150, 102	$C_5H_{11}NO_2S$	1.99	1	In-House Library
17	0.83	α-Aminoadipic acid	4.60	-0.01	-0.66	2.30	2.00E-12	4.34E-12	-	162.0761	161.0688	(+)162, 144, 97, 72, 84	$C_6H_{11}NO_4$	-1.91	2	HMDB
18	0.82	γ-Glutamylcysteine	1.53	0.00	-0.62	6.84	3.00E-11	7.41E-11	-	251.0696	250.0623	-	$C_8H_{14}N_2O_5S$	-1.61	1	In-House Library
19	0.84	Pyroglutamic acid	6.57	-0.02	-0.87	3.31	3.00E-29	2.12E-28	128.0355	130.0497	129.0426	(+)84, 130, 85, 102, 131	$C_5H_7NO_3$	1.45	1	In-House Library
20	1.03	N-Acetylglutamic acid	2.49	-0.01	-0.56	2.07	5.00E-09	9.43E-09	188.0564	190.071	189.0637	(+)130, 144, 84, 102	$C_7H_{11}NO_5$	4.37	2	Pubchem
21	1.03	Tyrosine	2.95	-0.01	-0.86	2.82	1.00E-23	6.01E-23	-	182.0808	181.0739	(+)136, 165, 123, 147, 119	$C_9H_{11}NO_3$	1.9	1	In-House Library
22	1.04	γ-Glutamylmethionine	1.84	-0.01	-0.53	9.59	1.00E-07	2.31E-07	277.0862	-	278.0935	(+)150, 133, 104, 116	$C_{10}H_{18}N_2O_5S$	-0.87	1	In-House Library
23	1.04	Isoleucine	18.12	-0.06	-0.90	3.84	2.00E-29	1.15E-28	-	132.1019	131.0946	(+)86, 132, 87, 45, 89	$C_6H_{13}NO_2$	-2.48	1	In-House Library
24	1.05	Phenylalanine	22.23	-0.07	-0.89	3.79	1.00E-27	7.85E-27	-	166.0859	165.079	(+)120, 166, 142, 84, 131	$C_9H_{11}NO_2$	-2.28	1	In-House Library
25	1.06	γ-Glutamylvaline	4.37	-0.01	-0.81	8.58	5.00E-22	1.91E-21	-	247.1284	246.1216	(+)201, 86, 132, 70, 247	$C_{10}H_{18}N_2O_5$	1.69	1	In-House Library
26	1.33	Tryptophan	8.60	-0.03	-0.85	3.72	1.00E-23	4.66E-23	-	205.0965	204.0899	(+)99, 188, 73, 146, 100	$C_{11}H_{12}N_2O_2$	-3.13	1	In-House Library
27	1.33	γ-Glutamylphenylalanine	0.81	0.00	-0.47	9.31	5.00E-06	6.63E-06	293.1142	-	294.1216	(-)293, 164, 128, 130, 127	$C_{14}H_{18}N_2O_5$	1.27	1	In-House Library
28	1.37	γ-Glutamylleucine	3.31	-0.01	-0.58	12.68	8.00E-09	1.55E-08	259.1299	261.1445	260.1372	(-)128, 130, 148, 197	$C_{11}H_{20}N_2O_5$	-0.59	1	In-House Library
29	5.3	γ-Glutamyltryptophan	0.37	0.00	-0.64	6.04	4.00E-11	8.62E-11		334.1395	333.1325	-	$C_{16}H_{19}N_3O_5$	2.35	1	In-House Library
30	5.71	N-Isovaleroylglycine	0.78	0.00	-0.53	4.23	2.00E-07	3.71E-07	158.0824	160.097	159.0897	(-)158, 97, 89, 114	$C_7H_{13}NO_3$	0.64	2	HMDB
31	5.8	Phenylacetylglycine	2.91	0.01	0.89	-6.11	2.00E-27	1.23E-26	192.0655	194.081	193.0739	(-)74, 102, 130, 147, 146	$C_{10}H_{11}NO_3$	-0.46	1	In-House Library
32	5.99	N-Acetylleucine	2.43	-0.01	-0.55	5.54	2.00E-08	4.68E-08	172.0979	174.1125	173.1052	(-)130, 172, 127, 171, 111	$C_8H_{15}NO_3$	0.7	2	HMDB
33	6.37	N-Acetylphenylalanine	1.83	-0.01	-0.66	5.36	2.00E-12	4.34E-12	206.0822	208.0967	207.0894	(-)162, 164, 206, 118, 91	$C_{11}H_{13}NO_3$	0.12	2	Pubchem
Carbohydrates																
34	0.75	Hexose phosphate	8.03	0.03	0.88	-2.64	9.00E-30	6.64E-29	259.0221	261.0363	260.0297	(-)96, 78, 259, 138, 241	$C_6H_{13}O_9P$	-0.99	1	In-House Library
35	0.75	Disaccharide	16.71	0.06	0.95	-2.64	1.00E-40	2.13E-39	341.1085	343.1226	342.1162	(+)85, 145, 91, 163, 127	$C_{12}H_{22}O_{11}$	-1.21	1	In-House Library
36	0.76	Hexose	1.74	0.01	0.10	-0.32	2.00E-01	1.99E-01	179.056	-	180.0634	(-)59, 89, 71, 113, 101	$C_6H_{12}O_6$	-0.54	2	MoNA
37	0.77	N-Acetylhexosamine phosphate	17.31	0.06	0.91	-9.96	2.00E-36	2.13E-35	300.0491	-	301.0563	(-)78, 300, 96, 199	$C_8H_{16}NO_9P$	-0.37	2	HMDB
38	0.78	Disaccharide phosphate	7.40	0.02	0.96	-4.37	5.00E-49	1.41E-47	421.0749	-	422.0825	(-)421, 241, 361, 96, 78	$C_{12}H_{23}O_{14}P$	0.82	2	HMDB
39	0.79	N-Acetylhexosamine	6.02	0.02	0.73	-3.38	1.00E-16	3.52E-16	-	222.0972	221.0899	(+)126, 138, 144, 168, 186	$C_8H_{15}NO_6$	-2.47	2	MoNA
Fatty acids & Lipids																
40	0.75	3-Hydroxymethylglutaric acid	10.73	0.04	0.97	-2.34	5.00E-47	1.02E-45	161.0456	163.0601	162.0528	(-)73, 59, 161, 131	$C_6H_{10}O_5$	0.29	2	Pubchem
41	0.75	Glycerophosphorylcholine	31.66	0.11	0.90	-2.31	1.00E-35	1.84E-34	-	258.1093	257.1028	(+)104, 258, 184, 124, 86	$C_8H_{20}NO_6P$	-3.16	2	MoNA
42	0.81	Aminovaleric acid betaine	6.85	0.02	0.92	-6.30	2.00E-37	2.38E-36	-	160.1328	159.1259	(+)160, 101, 60, 161, 55	$C_8H_{17}NO_2$	-2.36	2	MoNA
43	0.82	Propionylcarnitine	3.87	0.01	0.93	-3.89	3.00E-39	5.32E-38	-	218.1383	217.1314	(+)85, 218, 217, 159, 130	$C_{10}H_{19}NO_4$	-1.88	2	MoNA
44	0.83	α-Hydroxyglutaric acid	2.00	-0.01	-0.30	0.66	2.00E-03	2.82E-03	147.0299	-	148.0372	(-)129, 147, 103, 85, 101	$C_5H_8O_5$	0.07	1	In-House Library
45	1.04	Citraconic acid	3.28	0.01	0.73	-1.75	2.00E-16	5.05E-16	129.0193	-	130.0266	(-)85, 129, 128, 101, 86	$C_5H_6O_4$	0.48	1	In-House Library
46	1.05	Butyrylcarnitine	6.81	0.02	0.89	-7.64	8.00E-30	6.60E-29	-	232.1538	231.1471	(+)85, 232, 173, 60, 233	$C_{11}H_{21}NO_4$	-2.13	2	MoNA
47	1.07	Hydroxybutyric acid	2.02	0.01	0.79	-1.86	5.00E-19	1.55E-18	103.0401	-	104.0473	(-)103, 57, 102, 59, 104	$C_4H_8O_3$	0.84	2	MoNA
48	1.45	Valerylcarnitine	5.17	0.02	0.89	-10.24	1.00E-29	8.18E-29	-	246.1695	245.1627	(+)85, 245, 246, 187, 60	$C_{12}H_{23}NO_4$	-2.11	2	MoNA
49	2.09	2-Hydroxyisovaleric acid	2.05	-0.01	-0.24	1.08	2.00E-02	2.21E-02	117.0559	-	118.063	(-)116, 117, 99, 71, 115	$C_5H_{10}O_3$	1.56	1	In-House Library
50	5.63	2-Hydroxy-3-methylvaleric acid	1.29	0.00	0.06	-0.15	6.00E-01	6.48E-01	-	131.0715	132.0786	(-)131, 85, 132, 113, 86	$C_6H_{12}O_3$	1.38	1	In-House Library
51	5.76	2-Hydroxyisocaproic acid	4.08	-0.01	-0.25	1.07	1.00E-02	1.59E-02	131.0715	-	132.0786	(-)131, 85, 132, 113, 86	$C_6H_{12}O_3$	1.03	1	In-House Library
52	6.91	Azelaic acid	1.39	0.00	-0.38	0.70	7.00E-04	8.01E-04	187.0975	-	188.1049	(-)125, 187, 126, 186, 188	$C_9H_{16}O_4$	-0.26	2	MoNA
53	7.49	Decanedioic acid	1.75	-0.01	-0.53	1.92	4.00E-07	6.26E-07	201.1132	-	202.1205	(-)201, 139, 183, 99, 172	$C_{10}H_{18}O_4$	-0.36	2	MoNA
54	7.92	Taurocholic acid	0.67	0.00	0.55	-6.43	4.00E-07	5.97E-07	514.2839	-	515.292	(-)514, 515, 174, 311	$C_{26}H_{45}NO_7S$	0.29	1	In-House Library
55	8.01	Undecanedioic acid	1.26	0.00	-0.45	1.57	5.00E-05	6.77E-05	215.1289	-	216.1361	(-)215, 197, 153, 216, 198	$C_{11}H_{20}O_4$	0.04	2	Pubchem

56	8.2	TriHOME	6.28	-0.02	-0.42	5.70	1.00E-04	1.34E-04	329.2333	-	330.2406	(-)320, 212, 230, 171, 172	$C_{18}H_{34}O_5$	0.12	2	MoNA
57	8.28	Glycocholic acid	1.03	0.00	0.54	-5.29	7.00E-07	1.16E-06	466.3157	464.3013	465.309	(-)464, 465, 89, 74	$C_{26}H_{43}NO_6$	-0.4	1	In-House Library
58	8.5	Dodecanedioic acid	1.24	0.00	-0.45	1.68	5.00E-05	7.00E-05	229.1445	-	230.1518	(-)229, 211, 167, 116, 230	$C_{12}H_{22}O_4$	-0.01	1	In-House Library
59	8.66	Sphingosine	1.46	0.00	0.73	-0.72	3.00E-15	9.57E-15	-	300.2891	299.2824	(+)300, 301, 106, 88, 69	$C_{18}H_{37}NO_2$	2.05	2	MoNA
60	8.89	C17 Sphinganine	44.07	0.15	0.81	-0.69	8.00E-20	2.99E-19	-	288.2889	287.2824	(+)288, 289, 106, 270	$C_{17}H_{37}NO_2$	2.88	2	MoNA
61	9.1	Cholic acid	2.03	-0.01	-0.20	1.97	9.00E-02	9.60E-02	407.28	-	408.2876	(-)407, 59, 408, 347, 329, 61	$C_{24}H_{40}O_5$	-0.81	1	In-House Library
62	9.24	10-Hydroxydecanoic acid	0.81	0.00	0.52	-1.02	2.00E-07	3.96E-07	187.1339	-	188.1412	(-)187, 141, 125, 59, 142	$C_{10}H_{20}O_3$	-0.14	1	In-House Library
63	10.74	Linolenic acid	6.13	0.00	0.08	-0.34	5.00E-01	4.60E-01	-	279.2313	278.2246	(+)95, 81, 109, 147, 263	$C_{18}H_{30}O_2$	-1.25	2	MoNA
64	10.78	LPC 16:0	18.62	-0.06	-0.36	5.85	1.00E-03	1.30E-03	-	496.3388	495.3325	(+)184, 104, 496, 478, 86	$C_{24}H_{50}NO_7P$	-1.99	1	In-House Library
65	10.8	Linoleamide	22.42	0.06	0.63	-1.34	2.00E-10	4.93E-10	-	280.263	279.2562	(+)245, 263, 95, 81, 109	$C_{18}H_{33}NO$	1.87	2	HMDB
66	10.89	LPC 18:1	3.32	-0.01	-0.28	2.24	2.00E-02	1.71E-02	-	522.3547	521.3481	(+)184, 220, 104, 221, 185	$C_{26}H_{52}NO_7P$	-1.42	2	MoNA
67	10.98	9-HODE	4.70	-0.01	-0.33	4.82	4.00E-03	4.07E-03	295.2277	-	296.2349	(-)295, 277, 195, 59, 250	$C_{18}H_{32}O_3$	-0.08	2	HMDB
68	11.46	3-Ketosphingosine	22.99	0.03	0.27	-0.62	1.00E-02	1.30E-02	-	298.2734	297.2668	(+)97, 155, 83, 245, 95	$C_{18}H_{35}NO_2$	2.39	2	HMDB
69	11.87	Hydroxyoctadecanoic acid	11.77	-0.04	-0.59	3.27	8.00E-10	1.70E-09	299.2591	-	300.2664	(-)299, 300, 187, 281, 116	$C_{18}H_{36}O_3$	-0.35	2	Pubchem
70	11.88	Oleic acid	13.86	-0.04	-0.58	3.33	1.00E-09	2.45E-09	-	283.2627	282.2559	(+)57, 83, 97, 71, 135	$C_{18}H_{34}O_2$	1.74	2	HMDB
71	11.89	LPC 18:0	4.25	-0.01	-0.28	2.55	1.00E-02	1.53E-02	-	524.3708	523.3638	(+)184, 104, 524, 506, 86	$C_{26}H_{54}NO_7P$	-0.41	2	MoNA
72	14.21	Octadecanamide	62.30	0.19	0.71	-0.61	5.95E-14	1.57E-13	-	284.294	283.2875	(+)289, 88, 102, 57, 74	$C_{18}H_{37}NO$	2.66	2	MoNA
Organic acids																
73	0.78	Gluconic acid	5.40	-0.01	-0.40	2.61	9.00E-05	1.23E-04	195.0509	-	196.0581	(-)195, 75, 129, 99, 159	$C_6H_{12}O_7$	-0.39	2	Pubchem
74	0.8	N-Acetylneuraminic acid	3.69	-0.01	-0.31	0.82	2.00E-02	1.91E-02	308.0984	310.1125	309.106	(-)87, 170, 98, 119, 146	$C_{11}H_{19}NO_9$	-1.12	2	MoNA
75	0.81	Glyceric acid	2.60	0.01	0.43	-1.45	5.00E-05	6.45E-05	105.0193	-	106.0266	(-)105, 75, 59, 44, 56	$C_3H_6O_4$	0.88	1	In-House Library
76	0.82	Glutaconic acid	0.94	0.00	-0.10	0.19	5.00E-01	4.60E-01	129.0195	-	130.0266	(-)57, 67, 85, 129, 128	$C_5H_6O_4$	-0.59	2	HMDB
77	0.83	Pipecolic acid	3.26	-0.01	-0.35	1.57	1.00E-03	1.53E-03	-	130.0861	129.079	(+)130, 84, 131, 85, 70	$C_6H_{11}NO_2$	1.51	2	MoNA
78	0.83	Malic acid	6.28	-0.01	-0.28	1.30	1.00E-02	1.05E-02	133.0143	-	134.0215	(-)115, 133, 71, 132, 96	$C_4H_6O_5$	0.27	1	In-House Library
79	0.83	Orotic acid	11.76	0.04	0.97	-6.40	3.00E-52	1.38E-50	155.0098	-	156.0171	(-)111, 155, 112, 41	$C_5H_4N_2O_4$	-0.12	1	In-House Library
80	0.83	Uric acid	7.04	0.02	0.92	-8.48	1.00E-33	1.60E-32	167.021	169.0352	168.0283	(-)167, 124, 96, 149, 41	$C_5H_4N_4O_3$	-0.59	1	In-House Library
81	0.83	cis-Aconitic acid	1.03	0.00	0.08	-0.19	3.00E-01	3.02E-01	173.009	-	174.0164	(-)85, 111, 129, 173, 85	$C_6H_6O_6$	-0.65	1	In-House Library
82	0.83	Citric acid	17.60	-0.05	-0.33	1.86	5.00E-03	5.32E-03	191.0195	-	192.027	(-)111, 87, 85, 191, 129	$C_6H_8O_7$	-1.18	2	MoNA
83	0.83	Maleic acid	3.50	-0.01	-0.42	3.71	7.00E-05	9.54E-05	115.0038	117.0184	116.0111	(-)116, 115, 71, 100	$C_4H_4O_4$	1.42	1	In-House Library
84	0.88	Threonic acid	6.42	-0.02	-0.73	3.75	2.00E-15	6.82E-15	135.03	137.0446	136.0373	(-)135, 75, 89, 117	$C_4H_8O_5$	0.64	2	HMDB
85	0.93	α-Ketoglutaric acid	3.37	0.01	0.55	-1.73	1.00E-08	2.26E-08	145.0142	-	146.0215	(-)101, 100, 57, 145, 73	$C_5H_6O_5$	0.35	2	MoNA
86	1.03	Succinic acid	5.80	-0.02	-0.59	0.73	8.00E-10	1.63E-09	117.0193	-	118.0266	(-)116, 73, 117, 99, 115	$C_4H_6O_4$	0.66	1	In-House Library
87	1.03	Malonic acid	0.82	0.00	-0.37	5.89	7.00E-04	8.21E-04	103.0038	105.0184	104.011	(-)75, 103, 101, 59, 57	$C_3H_4O_4$	-1.71	2	HMDB
88	1.18	Ethyl acetoacetate	4.66	0.02	0.90	-1.14	4.00E-31	3.46E-30	-	131.07	130.063	(+)59, 84, 131, 45, 43	$C_6H_{10}O_3$	2.38	1	In-House Library
89	5.1	Hippuric acid	3.28	0.01	0.53	-7.16	2.00E-07	3.28E-07	178.051	180.0655	179.0582	(+)150, 174, 179, 160, 180	$C_9H_9NO_3$	-2.11	1	In-House Library
Benzenoids																
90	1.06	Phenylethylamine	2.04	-0.01	-0.29	3.87	4.00E-03	4.07E-03	-	122.0961	121.0891	(+)105, 122, 106, 121, 123	$C_8H_{11}N$	2.18	2	MoNA
91	5.81	Dihydroxybenzoic acid	1.14	0.00	0.47	-2.25	1.00E-05	1.71E-05	153.0194	-	154.0266	(-)153, 109, 135, 108, 154	$C_7H_6O_4$	0.5	1	In-House Library
Indoles																
92	1.11	Indole-3-acrylic acid	8.72	-0.03	-0.86	3.94	1.00E-24	5.83E-24	-	188.0706	187.0633	(+)146, 188, 144, 118, 170	$C_{11}H_9NO_2$	-2.12	1	In-House Library
93	5.03	Indole-3-carboxilic acid-O-sulfate	8.59	0.03	0.66	-13.36	2.00E-11	4.13E-11	239.9971	-	241.0045	(-)160, 239, 161, 241, 151	$C_9H_7NO_5S$	0.35	2	HMDB
94	6.58	Indole-3-lactic acid	2.90	-0.01	-0.41	1.26	8.00E-05	1.07E-04	204.0657	206.0809	205.0739	(-)204, 158, 186, 160, 116	$C_{11}H_{11}NO_3$	-0.36	2	MoNA
95	6.93	Indole-3-carboxaldehyde	0.44	0.00	-0.51	1.59	5.00E-07	8.43E-07	144.0457	-	145.0528	(-)100, 144, 115	C_9H_7ON	1.27	1	In-House Library
96	7.14	Indole-3-acetic acid	0.69	0.00	-0.29	4.82	1.00E-02	1.06E-02	-	176.0703	175.0633	(+)130, 176, 131, 171, 88	$C_{10}H_9O_2N$	-1.75	1	In-House Library
97	7.74	Indole-3-propionic acid	0.28	0.00	-0.40	4.59	5.00E-05	7.00E-05	-	190.086	189.079	(+)130, 144, 172, 190, 118	$C_{11}H_{11}O_2N$	-1.59	1	In-House Library
Nucleotides																
98	0.8	Adenosine phosphate	5.69	-0.02	-0.67	3.22	2.00E-12	4.34E-12	346.0556	348.0702	347.0629	(+)136, 137, 97, 348, 250	$C_{10}H_{14}N_5O_7P$	-2.16	2	Pubchem
99	0.82	Uridine phosphate	4.60	-0.01	-0.39	1.34	2.00E-04	2.41E-04	323.0284	325.0429	324.0357	(-)79, 96, 211, 323, 111	$C_9H_{13}N_2O_9P$	0.11	2	MoNA
Phenylpropanoic acids																
100	1.71	Hydroxyphenyllactic acid	3.75	0.01	0.50	-0.99	1.00E-06	1.67E-06	181.0508	-	182.0579	(-)163, 181, 135, 119, 72	$C_9H_{10}O_4$	-0.02	1	In-House Library
101	6.34	Phenyllactic acid	6.74	-0.02	-0.50	1.77	7.00E-07	1.15E-06	165.0558	-	166.063	(-)147, 165, 119, 72, 136	$C_9H_{10}O_3$	0.23	1	In-House Library
Vitamins																
102	0.83	Ascorbic acid	18.26	-0.06	-0.37	9.64	6.00E-04	7.73E-04	175.0247	177.0392	176.0319	(-)115, 175, 71, 87	$C_6H_8O_6$	-0.26	1	In-House Library
103	0.91	Nicotinic acid	5.64	-0.02	-0.57	4.41	5.00E-08	8.49E-08	-	124.039	123.032	(+)124, 123, 125, 80, 96	$C_6H_5NO_2$	2.15	2	MoNA
104	0.92	Niacinamide	3.48	-0.01	-0.44	3.51	5.00E-05	6.67E-05	-	123.0551	122.048	(+)123, 124, 80, 96, 79	$C_6H_6N_2O$	1.62	2	MoNA
105	1.04	Pantothenic acid	5.27	0.02	0.84	-2.61	2.00E-25	1.16E-24	-	220.1174	219.1107	(+)90, 203, 175, 202, 220	$C_9H_{17}NO_5$	-2.35	2	MoNA
106	5.41	Riboflavin	2.30	0.01	0.80	-3.66	3.00E-19	1.07E-18	-	377.1449	376.1383	(+)377, 243, 378, 359, 244	$C_{17}H_{20}N_4O_6$	-1.68	1	In-House Library
Etc.																
107	0.66	Histamine	0.41	0.00	-0.40	2.70	2.00E-05	2.86E-05	-	112.0869	111.0796	(+)112, 95, 88, 89, 99	$C_5H_9N_3$	0.15	2	MoNA
Non-identification																
108	0.64	N.I 1	7.49	-0.02	-0.38	5.73	2.00E-03	2.06E-03	272.9588	-	-	(-)158, 130, 159, 114, 216	-	-	-	-
109	0.74	N.I 2	13.47	0.04	0.73	-1.59	8.00E-14	2.11E-13	425.0694	381.0784 [d]	380.0712	(+)381, 382, 169, 142, 82	-	-	-	-
110	0.74	N.I 3	6.55	0.02	0.95	-5.57	3.00E-47	8.22E-46	556.1641	558.1786	557.1713	-	-	-	-	-
111	0.74	N.I 4	7.71	-0.02	-0.47	3.11	4.00E-06	6.59E-06	711.2195	-	-	-	-	-	-	-
112	0.74	N.I 5	11.97	-0.04	-0.57	2.53	1.00E-08	2.88E-08	549.1668	505.1759	504.1686	(+)161, 101, 179, 221, 383	-	-	-	-

113	0.75	N.I 6	8.09	-0.03	-0.76	2.49	1.00E-16	3.21E-16	488.1617	490.1763	489.169	(+)148, 130, 102, 84, 149	-	-	-	-
114	0.76	N.I 7	9.20	0.03	0.36	-0.27	8.00E-04	9.95E-04	387.114	-	-	(-)101, 89, 161, 179, 97	-	-	-	-
115	0.76	N.I 8	8.80	0.03	0.50	-0.80	2.00E-07	3.60E-07	683.2247	685.2392	684.2319	(+)163, 145, 85, 120, 97	-	-	-	-
116	0.77	N.I 9	7.80	0.03	0.96	-4.77	3.00E-51	1.13E-49	333.0462	-	-	-	-	-	-	-
117	0.77	N.I 10	12.84	0.04	0.90	-7.88	2.00E-32	1.66E-31	642.1649	644.1795	643.1722	(-)300, 78, 96, 301, 199	-	-	-	-
118	0.77	N.I 11	5.84	0.02	0.45	-2.27	3.00E-06	4.09E-06	277.033	279.0475	278.0403	(+)262, 260, 244, 216, 118	-	-	-	-
119	0.77	N.I 12	12.22	0.02	0.17	-0.31	2.00E-01	2.01E-01	439.0845	441.0991	440.0918	(+)98, 145, 85, 260, 127	-	-	-	-
120	0.78	N.I 13	9.05	0.03	0.87	-1.18	7.00E-26	3.40E-25	483.1112	485.1258	484.1185	(-)140, 78, 141, 228, 70	-	-	-	-
121	0.78	N.I 14	8.01	0.03	0.79	-2.24	1.00E-20	3.52E-20	140.996	143.0106	142.0033	(-)78, 140, 122, 96, 85	-	-	-	-
122	0.79	N.I 15	11.20	0.04	0.51	-1.87	3.00E-08	6.05E-08	215.0326	-	-	(-)89, 59, 71, 127, 215	-	-	-	-
123	0.79	N.I 16	6.47	0.02	0.50	-1.88	4.00E-08	6.66E-08	217.0297	-	-	-	-	-	-	-
124	0.79	N.I 17	13.27	0.04	0.42	-0.62	4.00E-05	6.11E-05	377.0852	-	-	(-)341, 179, 89, 161, 101	-	-	-	-
125	0.79	N.I 18	5.12	-0.02	-0.45	2.01	3.00E-05	3.76E-05	539.1358	-	-	(-)179, 89, 503, 161, 101	-	-	-	-
126	0.79	N.I 19	6.25	0.02	0.57	-1.45	5.00E-08	9.34E-08	194.9465	-	-	(-)96, 195, 75, 129, 99	-	-	-	-
127	0.8	N.I 20	7.45	0.02	0.87	-7.49	1.00E-27	7.85E-27	398.0267	-	-	-	-	-	-	-
128	0.8	N.I 21	7.61	0.03	0.75	-7.83	5.00E-17	1.57E-16	256.0592	-	-	(-)119, 101, 100 ,59, 89	-	-	-	-
129	0.82	N.I 22	6.90	0.02	0.51	-1.32	1.00E-07	2.46E-07	161.0456	163.0601	162.0528	(-)73, 59, 101, 71, 85	-	-	-	-
130	0.84	N.I 23	7.88	-0.02	-0.34	3.75	4.00E-04	5.43E-04	251.0773	253.0918	252.0845	(-)251, 89, 101, 119, 59	-	-	-	-
131	0.87	N.I 24	8.96	0.03	0.86	-4.57	3.00E-29	2.12E-28	199.8049	-	-	(-)199, 162, 164, 173, 155	-	-	-	-
132	0.87	N.I 25	12.84	0.04	0.85	-4.53	3.00E-28	1.45E-27	197.8079	-	-	(-)197, 162, 160, 178, 196	-	-	-	-
133	0.87	N.I 26	11.70	0.04	0.86	-4.58	1.00E-29	9.08E-29	195.811	-	-	(-)160, 195, 96, 178, 196	-	-	-	-
134	0.87	N.I 27	6.12	0.02	0.83	-1.39	5.00E-22	1.91E-21	134.8948	-	-	(-)134, 75, 89, 135, 116	-	-	-	-
135	1.04	N.I 28	8.29	0.03	0.72	-0.55	3.00E-14	7.51E-14	237.0532	239.0677	238.0604	(+)203, 175, 221, 204, 147	-	-	-	-
136	1.05	N.I 29	9.07	0.03	0.50	-10.29	1.00E-06	2.15E-06	360.0854	362.0999	361.0927	(+)278, 326, 344, 298, 200	-	-	-	-
137	5.54	N.I 30	6.10	0.02	0.75	-12.33	3.00E-15	8.17E-15	242.0128	244.0274	243.0201	(-)162, 242, 197, 241, 174	-	-	-	-
138	6.51	N.I 31	1.73	0.01	0.72	-11.34	1.00E-13	3.45E-13	-	421.2585	-	(+)89, 133, 177, 111, 99	-	-	-	-
139	6.66	N.I 32	15.30	-0.05	-0.81	9.57	9.00E-22	3.56E-21	-	628.3811	-	(+)133, 177, 155, 239	-	-	-	-
140	6.7	N.I 33	14.56	-0.05	-0.80	9.26	1.00E-21	3.71E-21	-	650.3951	-	(+)177, 155, 137, 199	-	-	-	-
141	9.45	N.I 34	6.84	0.02	0.65	-0.60	6.00E-12	1.41E-11	293.1758	-	-	(-)193, 236, 221, 249, 220	-	-	-	-
142	10.42	N.I 35	10.49	-0.03	-0.38	7.56	6.00E-04	7.81E-04	564.3304	-	-	(-)279, 280, 504, 224, 242	-	-	-	-
143	10.78	N.I 36	10.97	-0.04	-0.38	5.42	6.00E-04	7.73E-04	540.3303	-	-	(-)255, 480, 256, 224, 242	-	-	-	-
144	11.06	N.I 37	7.01	-0.02	-0.34	5.68	2.00E-03	2.47E-03	566.346	-	-	(-)281, 282, 506, 224, 242	-	-	-	-
145	11.62	N.I 38	7.03	-0.02	-0.40	10.01	3.00E-04	3.78E-04	537.3276	-	-	(-)255, 256, 253, 161, 101	-	-	-	-
146	11.88	N.I 39	6.40	-0.02	-0.57	3.41	4.00E-09	7.99E-09	399.1843	423.1808 [e]	400.1916	(-)116, 399, 381, 100, 363	-	-	-	-
147	13.76	N.I 40	12.85	-0.04	-0.60	0.87	2.00E-09	3.33E-09	355.1579	-	-	(-)355, 116, 311, 100, 84	-	-	-	-

[a] RT, Retention time

[b] VIP, Variable importance projection

[c] M.W., Molecular weight

[d] $[M\text{-}FA+H]^-$

[e] $[M+Na]^+$

Table S4. List of bioactive metabolites associated with probiotics or yogurt.

No.	Class	Compounds Name	Functional Properties [a]
1	**Amino acids**	**γ-Aminobutyric acid (GABA)**	Anti-hypertensive [1,2]
2		**Ornithine**	Stress reduction, Sleep-enhancing [3]
3		**α-Aminoadipic acid**	Anti-obesity [4]
4		**Betaine**	Cardiovascular benefits, Hepatoprotective [5]
5		**Creatine**	Anti-cancer, Anti-inflammatory, Neuroprotective, Muscle maintenance [6]
6		**Carnitine**	Antioxidant [7]
7		**Phenylacetylglycine**	Cardiovascular benefits [8]
8	**Peptides**	**γ-Glutamylvaline**	Anti-inflammatory [9]
9		**γ-Glutamylmethionine**	Anti-diabetic [10]
10		**γ-Glutamylleucine**	Anti-diabetic [10]
11		**γ-Glutamylphenylalanine**	Anti-diabetic [10]
12		**γ-Glutamylcysteine**	Anti-inflammatory [9]
13		**γ-Glutamyltryptophan**	Anti-diabetic [10]
14		**γ-Glutamyltyrosine**	Anti-diabetic [10]
15	**Fatty acids &**	**2-Hydroxyisovaleric acid**	Potential anti-diabetic [11]
16	**Lipids**	**2-Hydroxyisocaproic acid**	Potential anti-diabetic [11], Muscle mass gain [12]
17		**2-Hydroxy-3-methylvaleric acid**	Potential anti-diabetic [11]
18		**Acetate**	Metabolic regulation, Anti-inflammatory [13]
19		**Propionate**	Metabolic regulation, Anti-inflammatory [13]
20		**Butyrate**	Metabolic regulation, Anti-inflammatory [13]
21		**Cholic acid**	Anti-obesity [14]
22		**Glycocholic acid**	Anti-inflammatory [15]
23		**Taurocholic acid**	Anti-inflammatory [15]
24		**β-Hydroxybutyric acid**	Serum lipid profile improvement [16]
25		**β-hydroxy-β-methylbutyric acid**	Muscle-preserving [17]
26		**Glycerophosphorylcholine**	Antioxidant, Anti-inflammatory, Neuroprotective, Cognitive enhancing [18]
27		**3-Hydroxymethylglutaric acid**	Anti-cholesterolemic [19]
28	**Amines**	**Histamine**	Neuromodulator [1]
29		**Phenylethylamine**	Neuromodulator [20]
30		**Spermine**	Delay of brain aging [21]
31		**Spermidine**	Delay of brain aging [21]
32	**Organic acid**	**N-Acetylneuraminic acid**	Cardiovascular benefits [22]
33		**Orotic acid**	Hyperuricosuria relief, Microbial modulation [23]
34	**Benzenoids**	**3-Hydroxybenzoic acid**	Anti-Alzheimer's potential [24,25]
35		**3,4-Dihydroxybenzoic acid**	Anti-inflammatory [24]
36	**Indoles**	**Indole-3-carboxaldehyde**	Barrier-enhancing, Microbial modulation [26]
37		**Indole-3-propionic acid**	Barrier-enhancing [27], Anti-inflammatory, Microbial Modulation [28]
38		**Indole-3-acetic acid**	Barrier-enhancing, Immune-modulating [29]
39		**Indole-3-acrylic acid**	Barrier-enhancing, Immune-modulating [29], Antioxidant, Anti-inflammatory [30]

40		**Indole-3-lactic acid**	Barrier-enhancing, Immune-modulating [29]
41		**Indole-3-pyruvic acid**	Immune-modulating, Anti-inflammatory [27]
42		**Indole-3-acetamide**	Anti-inflammatory, Antioxidant [31]
43		**Tryptamine**	Regulating intestinal motility and immune [27]
44		**Indole**	Immune-modulating, Anti-inflammatory [27]
45		**Indoxyl-3-sulfate**	Anti-cancer [32]
46		**Indole-3-acetaldehyde**	Barrier-enhancing [33]
47	**Phenylpropanoids**	**Phenyllactic acid**	Antioxidant [34], Anti-ageing [35]
48		**Hydroxyphenyllactic acid**	Antioxidant, Gastrointestinal benefits [36]
49	**Vitamins**	**Retinol (Vitamin A)**	Antioxidant, Skin health, Vision, Immunity, Gene regulation [37]
50		**Ergocalciferol (Vitamin D)**	Regulation of calcium availability [37]
51		**α-Tocopherol (Vitamin E)**	Antioxidant, Cell membrane integrity, Immunity [37]
52		**Phylloquinone (Vitamin K1)**	Anti-inflammatory, Metabolic benefits [38]
53		**Menaquinone (Vitamin K2)**	Anti-diabetic [39]
54		**Thiamine (Vitamin B1)**	Cofactor of enzymes (cellular energetic metabolism) [37]
55		**Riboflavin (Vitamin B2)**	Antioxidant, Prosthetic group of enzymes, Oxidation-reduction, Cellular respiration, Energy metabolism [29,37]
56		**Nicotinic acid (Vitamin B3)**	Antioxidant, Cellular respiration, Energy metabolism [29,37]
57		**Niacinamide**	Neuroprotection, Skin health [40]
58		**Pantothenic acid (Vitamin B5)**	Cellular respiration, Energy metabolism, Transmission of nervous signal, Transport of blood gas [29,37]
59		**Pyridoxine (Vitamin B6)**	Transport of blood gas, Activation of other vitamins (B9 and B3) [37]
60		**Biotin (Vitamin B7)**	Prosthetic group of several enzymes, Energy metabolism, Regulation of gene expression [37]
61		**Folic acid (Vitamin B9)**	Hematopoiesis, Synthesis of nucleic base [37]
62		**Cobalamin (Vitamin B12)**	Hematopoiesis, Energy metabolism [37]
63		**Ascorbic acid (Vitamin C)**	Antioxidant, Cofactor of enzyme, Synthesis of hormones and neurotransmitters [29,37]
64		**Dehydroascorbic acid**	Antiviral effects [41]
65	**Pyrimidine**	**Uridine**	Gastrointestinal benefits [42]

[a] Functional properties were described based on previously reported literature.

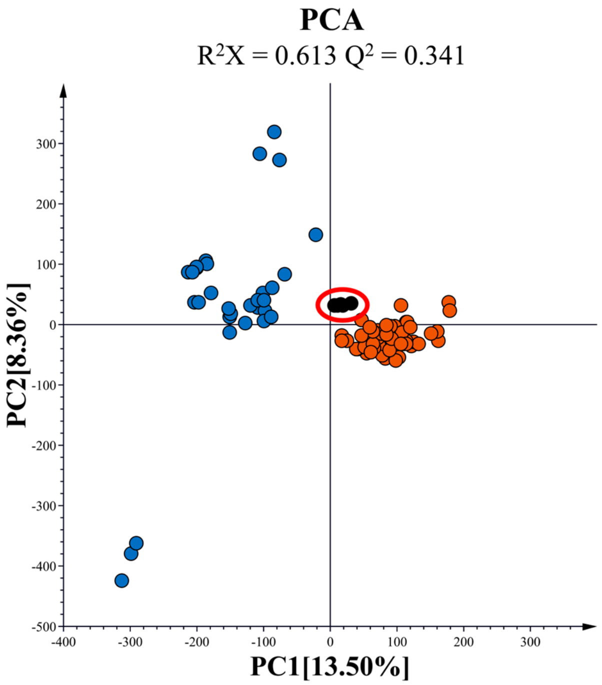

Fig. S1. Principal component analysis (PCA) score plots in integrated positive and negative ionization modes between probiotic supplements (blue circles), yogurt (orange circles) and quality control samples (black circles) based on UHPLC–Orbitrap-MS/MS.

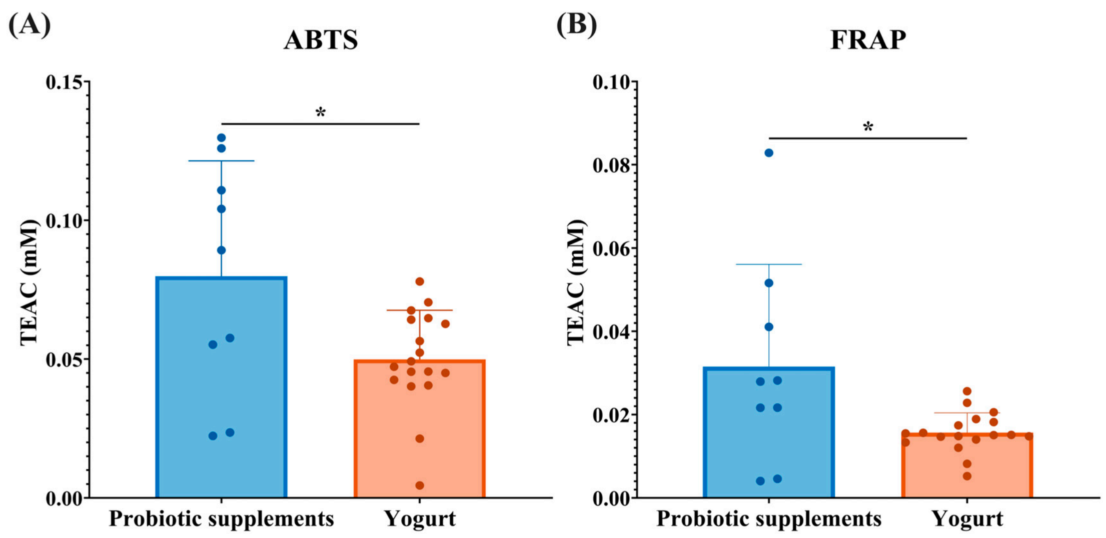

Fig. S2. Antioxidant assays after exclusion of two samples with extreme ABTS and FRAP values in probiotic supplements (PS) and yogurt (YG). (A) ABTS radical scavenging assay; (B) ferric reducing antioxidant power (FRAP) assay. The y-axis in (A) and (B) represents Trolox equivalent antioxidant capacity (TEAC). Statistical significance is indicated by asterisks (*$p < 0.05$).

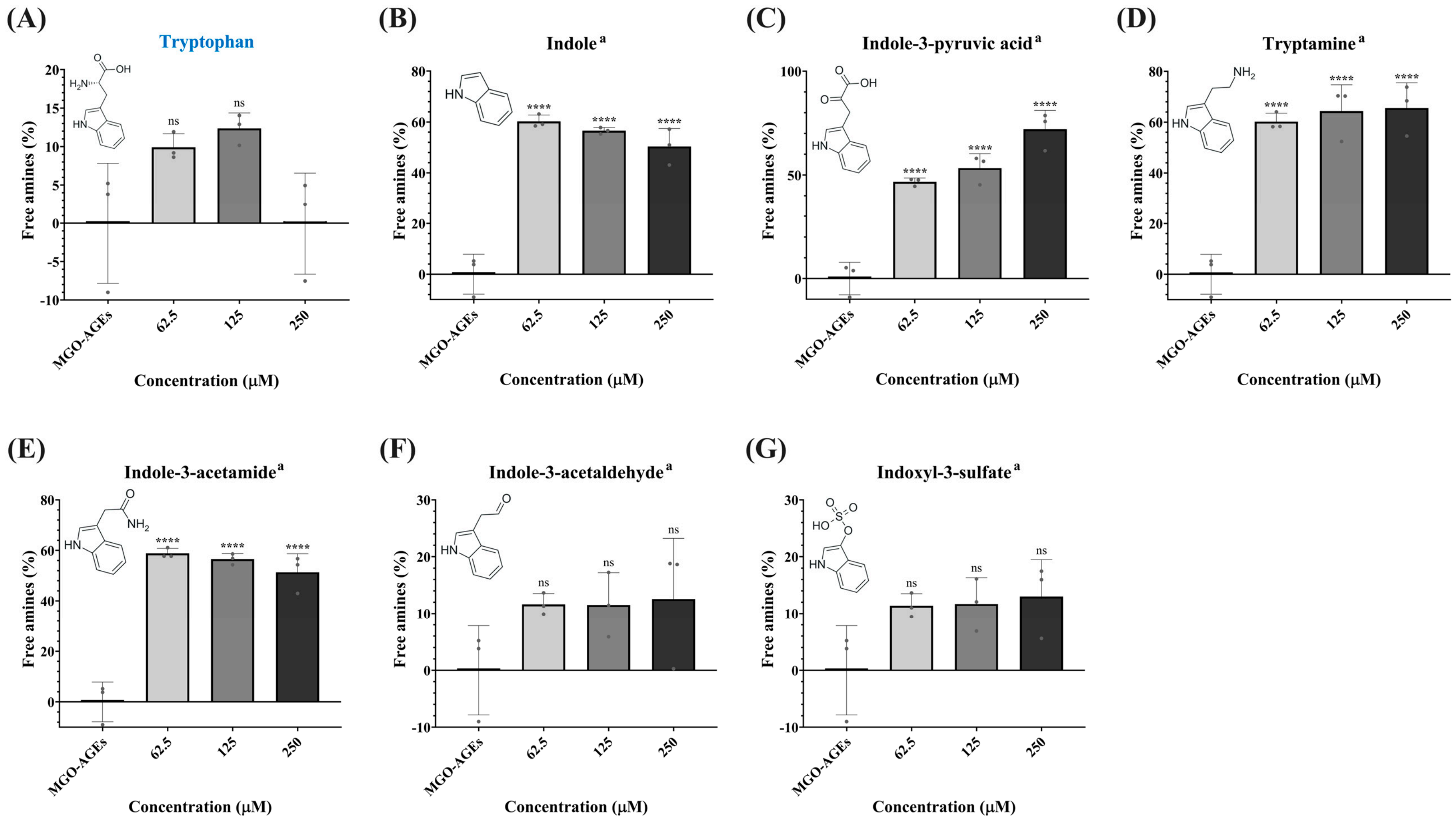

Fig. S3. Antiglycation activity of tryptophan and indole derivatives in the MGO–AGEs breaking assay. (A) Tryptophan; (B) indole; (C) indole-3-pyruvic acid; (D) tryptamine; (E) indole-3-acetamide; (F) indole-3-acetaldehyde; and (G) indoxyl-3-sulfate. PS-specific marker metabolites determined in Fig. 3 are shown in blue text. Bioactive metabolites are denoted by the letter (a), and detailed information is available in Table S4. Results are presented as mean ± SD of technical replicates (n = 3). ****$p < 0.0001$ vs. MGO–AGEs (control); ns indicates not significant ($p > 0.05$).

Reference

1. Diez-Gutiérrez, L.; San Vicente, L.; R. Barrón, L.J.; Villarán, M. del C.; Chávarri, M. Gamma-Aminobutyric Acid and Probiotics: Multiple Health Benefits and Their Future in the Global Functional Food and Nutraceuticals Market. *J. Funct. Foods* **2020**, *64*, 103669, doi:10.1016/j.jff.2019.103669.

2. Inoue, K.; Shirai, T.; Ochiai, H.; Kasao, M.; Hayakawa, K.; Kimura, M.; Sansawa, H. Blood-Pressure-Lowering Effect of a Novel Fermented Milk Containing γ-Aminobutyric Acid (GABA) in Mild Hypertensives. *Eur. J. Clin. Nutr.* **2003**, *57*, 490–495, doi:10.1038/sj.ejcn.1601555.

3. Miyake, M.; Kirisako, T.; Kokubo, T.; Miura, Y.; Morishita, K.; Okamura, H.; Tsuda, A. Randomised Controlled Trial of the Effects of L-Ornithine on Stress Markers and Sleep Quality in Healthy Workers. *Nutr. J.* **2014**, *13*, 53, doi:10.1186/1475-2891-13-53.

4. Xu, W.-Y.; Shen, Y.; Zhu, H.; Gao, J.; Zhang, C.; Tang, L.; Lu, S.-Y.; Shen, C.-L.; Zhang, H.-X.; Li, Z.; et al. 2-Aminoadipic Acid Protects against Obesity and Diabetes. *Journal of Endocrinology* **2019**, *243*, 111–123, doi:10.1530/JOE-19-0157.

5. Craig, S.A. Betaine in Human Nutrition. *Am. J. Clin. Nutr.* **2004**, *80*, 539–549, doi:10.1093/ajcn/80.3.539.

6. Kreider, R.B.; Stout, J.R. Creatine in Health and Disease. *Nutrients* **2021**, *13*, 447, doi:10.3390/nu13020447.

7. Gülçin, İ. Antioxidant and Antiradical Activities of L-Carnitine. *Life Sci.* **2006**, *78*, 803–811, doi:10.1016/j.lfs.2005.05.103.

8. Xu, X.; Lu, W.; Shi, J.; Su, Y.; Liu, Y.; Wang, L.; Xiao, C.; Chen, C.; Lu, Q. The Gut Microbial Metabolite Phenylacetylglycine Protects against Cardiac Injury Caused by Ischemia/Reperfusion through Activating B2AR. *Arch. Biochem. Biophys.* **2021**, *697*, 108720, doi:10.1016/j.abb.2020.108720.

9. Zhang, H.; Kovacs-Nolan, J.; Kodera, T.; Eto, Y.; Mine, Y. γ-Glutamyl Cysteine and γ-Glutamyl Valine Inhibit TNF-α Signaling in Intestinal Epithelial Cells and Reduce Inflammation in a Mouse Model of Colitis via Allosteric Activation of the Calcium-Sensing Receptor. *Biochimica et Biophysica Acta (BBA) - Molecular Basis of Disease* **2015**, *1852*, 792–804, doi:10.1016/j.bbadis.2014.12.023.

10. Yang, J.; Sun-Waterhouse, D.; Cui, C.; Dong, K.; Zhao, M. *γ* -Glu-Met Synthesised Using a Bacterial Glutaminase as a Potential Inhibitor of Dipeptidyl Peptidase IV. *Int. J. Food Sci. Technol.* **2018**, *53*, 1166–1175, doi:10.1111/ijfs.13692.

11. Daniel, N.; Nachbar, R.T.; Tran, T.T.T.; Ouellette, A.; Varin, T.V.; Cotillard, A.; Quinquis, L.; Gagné, A.; St-Pierre, P.; Trottier, J.; et al. Gut Microbiota and Fermentation-Derived Branched Chain Hydroxy Acids Mediate Health Benefits of Yogurt Consumption in Obese Mice. *Nat. Commun.* **2022**, *13*, 1343, doi:10.1038/s41467-022-29005-0.

12. Mero, A.A.; Ojala, T.; Hulmi, J.J.; Puurtinen, R.; Karila, T.A.; Seppälä, T. Effects of Alfa-Hydroxy-Isocaproic Acid on Body Composition, DOMS and Performance in Athletes. *J. Int. Soc. Sports Nutr.* **2010**, *7*, 1–8, doi:10.1186/1550-2783-7-1.

13. Canfora, E.E.; Jocken, J.W.; Blaak, E.E. Short-Chain Fatty Acids in Control of Body Weight and Insulin Sensitivity. *Nat. Rev. Endocrinol.* **2015**, *11*,

577–591, doi:10.1038/nrendo.2015.128.

14. Ikemoto, S.; Takahashi, M.; Tsunoda, N.; Maruyama, K.; Itakura, H.; Kawanaka, K.; Tabata, I.; Higuchi, M.; Tange, T.; Yamamoto, T.T. Cholate Inhibits High-Fat Diet-Induced Hyperglycemia and Obesity with Acyl-CoA Synthetase MRNA Decrease. *American Journal of Physiology-Endocrinology and Metabolism* **1997**, *273*, E37–E45, doi:https://doi.org/10.1152/ajpendo.1997.273.1.E37.

15. Ge, X.; Huang, S.; Ren, C.; Zhao, L. Taurocholic Acid and Glycocholic Acid Inhibit Inflammation and Activate Farnesoid X Receptor Expression in LPS-Stimulated Zebrafish and Macrophages. *Molecules* **2023**, *28*, 2005, doi:10.3390/molecules28052005.

16. Caminhotto, R. de O.; Komino, A.C.M.; de Fatima Silva, F.; Andreotti, S.; Sertié, R.A.L.; Boltes Reis, G.; Lima, F.B. Oral β-Hydroxybutyrate Increases Ketonemia, Decreases Visceral Adipocyte Volume and Improves Serum Lipid Profile in Wistar Rats. *Nutr. Metab. (Lond).* **2017**, *14*, 31, doi:10.1186/s12986-017-0184-4.

17. Deutz, N.E.P.; Pereira, S.L.; Hays, N.P.; Oliver, J.S.; Edens, N.K.; Evans, C.M.; Wolfe, R.R. Effect of β-Hydroxy-β-Methylbutyrate (HMB) on Lean Body Mass during 10 Days of Bed Rest in Older Adults. *Clinical Nutrition* **2013**, *32*, 704–712, doi:10.1016/j.clnu.2013.02.011.

18. Rafique, N.; Jan, S.Y.; Dar, A.H.; Dash, K.K.; Sarkar, A.; Shams, R.; Pandey, V.K.; Khan, S.A.; Amin, Q.A.; Hussain, S.Z. Promising Bioactivities of Postbiotics: A Comprehensive Review. *J. Agric. Food Res.* **2023**, *14*, 100708, doi:10.1016/j.jafr.2023.100708.

19. Di Padova, C.; Bosisio, E.; Cighetti, G.; Rovagnati, P.; Mazzocchi, M.; Colombo, C.; Tritapepe, R. 3-Hydroxy-3-Methylglutaric Acid (HMGA) Reduces Dietary Cholesterol Induction of Saturated Bile in Hamster. *Life Sci.* **1982**, *30*, 1907–1914, doi:10.1016/0024-3205(82)90471-4.

20. Shimazu, S.; Miklya, I. Pharmacological Studies with Endogenous Enhancer Substances: β-Phenylethylamine, Tryptamine, and Their Synthetic Derivatives. *Prog. Neuropsychopharmacol. Biol. Psychiatry* **2004**, *28*, 421–427, doi:10.1016/j.pnpbp.2003.11.016.

21. Xu, T.-T.; Li, H.; Dai, Z.; Lau, G.K.; Li, B.-Y.; Zhu, W.-L.; Liu, X.-Q.; Liu, H.-F.; Cai, W.-W.; Huang, S.-Q.; et al. Spermidine and Spermine Delay Brain Aging by Inducing Autophagy in SAMP8 Mice. *Aging* **2020**, *12*, 6401–6414, doi:10.18632/aging.103035.

22. Guo, S.; Tian, H.; Dong, R.; Yang, N.; Zhang, Y.; Yao, S.; Li, Y.; Zhou, Y.; Si, Y.; Qin, S. Exogenous Supplement of N-Acetylneuraminic Acid Ameliorates Atherosclerosis in Apolipoprotein E-Deficient Mice. *Atherosclerosis* **2016**, *251*, 183–191, doi:10.1016/j.atherosclerosis.2016.05.032.

23. Löffler, M.; Carrey, E.A.; Zameitat, E. Orotic Acid, More Than Just an Intermediate of Pyrimidine de Novo Synthesis. *Journal of Genetics and Genomics* **2015**, *42*, 207–219, doi:10.1016/j.jgg.2015.04.001.

24. Kiriyama, Y.; Tokumaru, H.; Sadamoto, H.; Kobayashi, S.; Nochi, H. Effects of Phenolic Acids Produced from Food-Derived Flavonoids and Amino Acids by the Gut Microbiota on Health and Disease. *Molecules* **2024**, *29*, 5102, doi:10.3390/molecules29215102.

25. Pasinetti, G. Synbiotic-Derived Metabolites Reduce Neuroinflammatory Symptoms of Alzheimer's Disease. *Curr. Dev. Nutr.* **2020**, *4*, nzaa062_035, doi:10.1093/cdn/nzaa062_035.

26. Zhang, R.; Huang, G.; Ren, Y.; Wang, H.; Ye, Y.; Guo, J.; Wang, M.; Zhu, W.; Yu, K. Effects of Dietary Indole-3-Carboxaldehyde Supplementation on Growth Performance, Intestinal Epithelial Function, and Intestinal Microbial Composition in Weaned Piglets. *Front. Nutr.* **2022**, *9*, 896815, doi:10.3389/fnut.2022.896815.

27. Ye, X.; Li, H.; Anjum, K.; Zhong, X.; Miao, S.; Zheng, G.; Liu, W.; Li, L. Dual Role of Indoles Derived from Intestinal Microbiota on Human Health. *Front. Immunol.* **2022**, *13*, 903526, doi:10.3389/fimmu.2022.903526.

28. Zhao, Z.-H.; Xin, F.-Z.; Xue, Y.; Hu, Z.; Han, Y.; Ma, F.; Zhou, D.; Liu, X.-L.; Cui, A.; Liu, Z.; et al. Indole-3-Propionic Acid Inhibits Gut Dysbiosis and Endotoxin Leakage to Attenuate Steatohepatitis in Rats. *Exp. Mol. Med.* **2019**, *51*, 1–14, doi:10.1038/s12276-019-0304-5.

29. Peluzio, M. do C.G.; Martinez, J.A.; Milagro, F.I. Postbiotics: Metabolites and Mechanisms Involved in Microbiota-Host Interactions. *Trends Food Sci. Technol.* **2021**, *108*, 11–26, doi:10.1016/j.tifs.2020.12.004.

30. Zhang, Q.; Wang, Y.; Wang, Y.; Yuan, J.; Wang, Y.; Zeng, Y.; Zhang, H.; Yang, H.; Ma, Q.; Shi, D. Effects of 3-Indoleacrylic Acid on Alleviating Lipopolysaccharide-Induced Liver Inflammatory Damage in Laying Hens. *Poult. Sci.* **2025**, 105307, doi:10.1016/j.psj.2025.105307.

31. Wang, M.; Feng, X.; Zhao, Y.; Lan, Y.; Xu, H. Indole-3-Acetamide from Gut Microbiota Activated Hepatic AhR and Mediated the Remission Effect of Lactiplantibacillus Plantarum P101 on Alcoholic Liver Injury in Mice. *Food Funct.* **2023**, *14*, 10535–10548, doi:10.1039/D3FO03585A.

32. Dalal, N.; Makharia, G.K.; Dalal, M.; Mohan, A.; Singh, R.; Kumar, A. Gut Metabolite Indoxyl Sulfate Has Selective Deleterious and Anticancer Effect on Colon Cancer Cells. *J. Med. Chem.* **2023**, *66*, 17074–17085, doi:10.1021/acs.jmedchem.3c01907.

33. Song, Y.; Zhang, J.; Liang, S.; Cao, Y.; Wang, Q. Hydroxytyrosol Gut Microbial Metabolites Promote Tight Junction Protein Expression via the AhR-Nrf2 Pathway. *Journal of Future Foods* **2025**, doi:10.1016/j.jfutfo.2025.10.014.

34. Beloborodova, N.; Bairamov, I.; Olenin, A.; Shubina, V.; Teplova, V.; Fedotcheva, N. Effect of Phenolic Acids of Microbial Origin on Production of Reactive Oxygen Species in Mitochondria and Neutrophils. *J. Biomed. Sci.* **2012**, *19*, 89, doi:10.1186/1423-0127-19-89.

35. Kim, J.; Jo, Y.; Lim, G.; Ji, Y.; Roh, J.-H.; Kim, W.-G.; Yi, H.-S.; Choi, D.W.; Cho, D.; Ryu, D. A Microbiota-Derived Metabolite, 3-Phenyllactic Acid, Prolongs Healthspan by Enhancing Mitochondrial Function and Stress Resilience via SKN-1/ATFS-1 in C. Elegans. *Nat. Commun.* **2024**, *15*, 10773, doi:10.1038/s41467-024-55015-1.

36. González, A.; Gálvez, N.; Martín, J.; Reyes, F.; Pérez-Victoria, I.; Dominguez-Vera, J.M. Identification of the Key Excreted Molecule by Lactobacillus Fermentum Related to Host Iron Absorption. *Food Chem.* **2017**, *228*, 374–380, doi:10.1016/j.foodchem.2017.02.008.

37. Gaucheron, F. Milk and Dairy Products: A Unique Micronutrient Combination. *J. Am. Coll. Nutr.* **2011**, *30*, 400S-409S, doi:10.1080/07315724.2011.10719983.

38. Juanola-Falgarona, M.; Salas-Salvadó, J.; Estruch, R.; Portillo, M.P.; Casas, R.; Miranda, J.; Martínez-González, M.A.; Bulló, M. Association between Dietary Phylloquinone Intake and Peripheral Metabolic Risk Markers Related to Insulin Resistance and Diabetes in Elderly Subjects at

High Cardiovascular Risk. *Cardiovasc. Diabetol.* **2013**, *12*, 7, doi:10.1186/1475-2840-12-7.

39. Li, Y.; Chen, J. peng; Duan, L.; Li, S. Effect of Vitamin K2 on Type 2 Diabetes Mellitus: A Review. *Diabetes Res. Clin. Pract.* **2018**, *136*, 39–51, doi:10.1016/j.diabres.2017.11.020.

40. Hwang, E.S.; Song, S.B. Possible Adverse Effects of High-Dose Nicotinamide: Mechanisms and Safety Assessment. *Biomolecules* **2020**, *10*, 687, doi:10.3390/biom10050687.

41. UOZAKI, M.; IKEDA, K.; TSUJIMOTO, K.; NISHIDE, M.; YAMASAKI, H.; KHAMSRI, B.; KOYAMA, A.H. Antiviral Effects of Dehydroascorbic Acid. *Exp. Ther. Med.* **2010**, *1*, 983–986, doi:10.3892/etm.2010.139.

42. Liu, Y.; Zhang, Y.; Yin, J.; Ruan, Z.; Wu, X.; Yin, Y. Uridine Dynamic Administration Affects Circadian Variations in Lipid Metabolisms in the Liver of High-Fat-Diet-Fed Mice. *Chronobiol. Int.* **2019**, *36*, 1258–1267, doi:10.1080/07420528.2019.1637347.
